# The Guaymas Basin Hiking Guide to Hydrothermal Mounds, Chimneys, and Microbial Mats: Complex Seafloor Expressions of Subsurface Hydrothermal Circulation

**DOI:** 10.3389/fmicb.2016.00075

**Published:** 2016-02-18

**Authors:** Andreas Teske, Dirk de Beer, Luke J. McKay, Margaret K. Tivey, Jennifer F. Biddle, Daniel Hoer, Karen G. Lloyd, Mark A. Lever, Hans Røy, Daniel B. Albert, Howard P. Mendlovitz, Barbara J. MacGregor

**Affiliations:** ^1^Department of Marine Sciences, University of North Carolina at Chapel HillChapel Hill, NC, USA; ^2^Microsensor Group, Max Planck Institute for Marine MicrobiologyBremen, Germany; ^3^Center for Biofilm Engineering and Department of Land Resources and Environmental Sciences, Montana State UniversityBozeman, MT, USA; ^4^Department of Marine Chemistry and Geochemistry, Woods Hole Oceanographic InstitutionWoods Hole, MA, USA; ^5^School of Marine Science and Policy, University of DelawareLewes, DE, USA; ^6^Department of Microbiology, University of Tennessee at KnoxvilleKnoxville, TN, USA; ^7^Department of Environmental Sciences, Eidgenössische Technische HochschuleZurich, Switzerland; ^8^Center for Geomicrobiology, Aarhus UniversityAarhus, Denmark

**Keywords:** Guaymas basin, hydrothermal circulation, hydrothermal sediment, *Beggiatoa* mat, *in situ* profiles, heatflow, porewater chemistry

## Abstract

The hydrothermal mats, mounds, and chimneys of the southern Guaymas Basin are the surface expression of complex subsurface hydrothermal circulation patterns. In this overview, we document the most frequently visited features of this hydrothermal area with photographs, temperature measurements, and selected geochemical data; many of these distinct habitats await characterization of their microbial communities and activities. Microprofiler deployments on microbial mats and hydrothermal sediments show their steep geochemical and thermal gradients at millimeter-scale vertical resolution. Mapping these hydrothermal features and sampling locations within the southern Guaymas Basin suggest linkages to underlying shallow sills and heat flow gradients. Recognizing the inherent spatial limitations of much current Guaymas Basin sampling calls for comprehensive surveys of the wider spreading region.

## Introduction

The Guaymas Basin in the Gulf of California is a young marginal rift basin characterized by active seafloor spreading and rapid deposition of organic-rich, diatomaceous sediments from highly productive overlying waters (Calvert, 1966). Organic-rich sediments of several hundred meters thickness overlie the spreading centers of Guaymas Basin and alternate with shallow intrusions of doleritic sills into the unconsolidated sediments (Einsele et al., 1980; Saunders et al., 1982). These magmatic intrusions into sediments produce organically derived thermogenic alteration products dominated by methane (Whelan et al., 1988), CO_2_, low-molecular weight organic acids (Martens, 1990), ammonia (Von Damm et al., 1985), and a wide spectrum of hydrocarbons (Simoneit and Lonsdale, 1982; Simoneit, 1985; Bazylinski et al., 1988; Whelan et al., 1988) that are released into sedimentary pore fluid and the ocean. Organic-rich fluids transported to the upper sediment column provide fossil carbon substrates to highly active, benthic microbial communities that oxidize and assimilate them (Pearson et al., 2005; Kniemeyer et al., 2007; Teske et al., 2014).

The two (northern and southern) axial troughs of Guaymas Basin are bounded by extensive systems of axial-parallel fault lines on both sides (Lonsdale and Becker, 1985; Fisher and Becker, 1991). Active hydrothermalism is predominantly found in the southern trough, where the hydrothermal sediments, mounds and chimneys form a complex hydrothermal landscape on the seafloor (Lonsdale and Becker, 1985). The conspicuous diversity of these seafloor features reflects different geochemical and temperature settings; their hydrothermal reactions, driven by underlying thermodynamic disequilibria, are modulated by location-specific reaction pathways in deep sediments or volcanic sills, and the variable residence times of hydrothermal liquid following these reaction pathways and hydrothermal circulation patterns (Gieskes et al., 1982; Kastner, 1982). Hydrothermal reactions generate and mobilize volatile hydrocarbons that migrate to the sediment surface (Peter et al., 1991; Lizarralde et al., 2011), under spatiotemporally variable temperature regimes that may limit or favor biological oxidation and assimilation (Biddle et al., 2012; McKay et al., 2012). This subsurface processing system produces a maze of subsurface flow pathways that ultimately reach the sediment surface, where they are evident in hydrothermal edifices of different developmental stages, hydrothermal mineral deposits, venting orifices emitting hot hydrothermal fluids, and hydrothermally active sediments.

The complex hydrothermal features at the Guaymas Basin seafloor were previously mapped and to a limited extent photographically documented, using a combination of dives with research submersible HOV *Alvin*, deep tow sonar records, and deep tow thermistor measurements (Koski et al., 1985; Lonsdale and Becker, 1985; Peter and Scott, 1988). The black and white photographs published in these early surveys permitted the first glimpses of the Guaymas Basin hydrothermal vent environment. Color images of hydrothermally active sediments with microbial mats and *Riftia* colonies were taken from HOV *Alvin*, and showed the distinct white, yellow, and orange coloration of the microbial mats, dominated by large filamentous sulfur-oxidizing bacteria, at that time ascribed to the genus *Beggiatoa* (Jannasch et al., 1989; Gundersen et al., 1992). Benthic communities and microbial mats revealing off-axis hydrothermal influence on the sedimented ridge flanks of Guaymas Basin were recorded by deep tow color photography (Lizarralde et al., 2011). Recent photos of well-documented Guaymas Basin hydrothermal sediments and microbial mats have accompanied published papers in online Supplementary Material (Holler et al., 2011; McKay et al., 2012), sometimes in edited form to show the location of temperature measurements (McKay et al., 2012), or they have appeared as small-scale figure inserts to illustrate sampling site context (Callac et al., 2013; MacGregor et al., 2013b). To our knowledge, only two publications have made an effort to document at least some of multiple Guaymas Basin sampling sites in color figures specifically for this purpose (Lizarralde et al., 2011; Meyer et al., 2013).

In contrast to the limited and widely scattered published image material, research cruises and individual cruise participants often accumulate a surprising amount of *in situ* observations and high-definition camera images, with varying degrees of scientific context and auxiliary data. Although these images provide the closest approximation of the *in situ* aspect of remote and rarely visited deep-sea hydrothermal vent environments, their context with respect to precise location, time, and observations linked to the site gets lost without detailed curation and documentation; all too often these important resources remain underused and serve merely as “decoration” for cruise blogs and occasional lectures. Here, we place detailed and previously unpublished *in situ* photographic surveys of microbial mats, hydrothermal mounds and chimneys collected by HOV *Alvin* during two cruises to the southern Guaymas Basin spreading center (AT15-40 and AT15-56) into the context of location, time, *in situ* data, and published studies that add to the thermal and geochemical site characterization (**Table 1**). In particular, *in situ* microprofiler deployments at several locations provide highly resolved chemical gradients at the sediment–water interface, where coinciding electron donors and acceptors provide energy for microbial mat growth.

**Table 1:** Site compilation including sampling or measurement locations, latitude/longitude, dive context, and relevant publications by the AT15-40 and AT15-56 science crew.

Site and Figure	Latitude and Longitude	Alvin Dive and Cruise Number, and localization context	Reference
**Microbial mats**			
Marker 2 mat, **Figure 2A**	27°00.468 N, 111°24.537 W	4483; AT15-40 shipboard xy fix	This publication
Marker 14 mat, **Figure 2B**	27°00.470 N, 111°24.431 W	4562; AT15-56 dive target position	McKay et al., 2012, 2015
Marker 27 mat, **Figure 2C**	27°00.445 N, 111°24.529 W	4564, 4572; AT15-56 dive target position	Bowles et al., 2012, McKay et al., 2012
Survey site 2 mat, **Figure 3A**	27°00.403 N, 111°24.459 W	4492; AT15-40 shipboard xy fix	Meyer et al., 2013
Japan-shaped mat near Mat Mound, **Figure 3B**	27°00.379 N; 111°24.566 W	4493; AT15-40 shipboard xy fix	This publication
UNC Mat, **Figure 3C**	27°00.445 N, 111°24.530 W	4489; AT15-40 shipboard xy fixes	Biddle et al., 2012, Ruff et al., 2015^∗^
Marker 6 mat, **Figure 4**	27°00.423 N, 111°24.477 W	4484, 4562; AT15-40 shipboard xy fix	This publication
Megamat, **Figure 5A**	27°00.464 N, 111°24.512 W	4485, 4486, 4488, 4490, 4491, 4562; AT15-40 shipboard xy fix	Biddle et al., 2012, McKay et al., 2012, Ruff et al., 2015^∗^
Cathedral Hill/Marker 24, **Figure 5B**	27°00.696 N, 111°24.265 W	4565; AT15-56 dive target position	This publication
Temperate mat, **Figure 5C**	27°00.786 N, 111°24.612 W	4574; AT15-56 frame grabber coordinates	This publication
**Microprofiler sites**			
Orange mat near Marker14, **Figure 6**	27°00.466 N, 111°24.425 W	4564; AT15-56 frame grabber coordinates	This publication
“AcetoBalsamico” mat near Marker 14, **Figure 7**	27°00.470 N, 111°24.427 W	4562, 4569, 4570, 4573; AT15-56 frame grabber coordinates	This publication
Hot profiler site near Marker 27, **Figures 8A,B**	27°00.448 N, 111°24.541 W	4566, 4567; AT15-56 frame grabber coordinates	This publication
Background sediment profiler site, **Figures 8C,D**	27°00.436 N, 111°24.480 W	4569; AT15-56 frame grabber coordinates	This publication
**Hydrothermal structures**			
Big Pagoda, **Figure 9**	27°00.909 N, 111°24.639 W	4574; AT15-56 frame grabber coordinates	This publication
Robins Roost, **Figure 10**	27°00.833 N, 111°24.679 W	4574; AT15-56 frame grabber coordinates	This publication
Rebecca’s Roost, **Figure 11**	27°00.683 N; 111°24.404 W	4574; AT15-56 frame grabber coordinates	This publication
Busted Mushroom, **Figure 12**	27°00.63 N, 111°24.41 W	4555; 4557; 4571; AT15-56 dive target position	Pagé et al., 2008
Mat Mound, **Figure 13**	27°00.388 N, 111°24.560 W	4483, 4484; AT15-40 shipboard xy fix	Ruff et al., 2015^∗^, Dowell et al., 2016
Wonder Mound, **Figure 14**	27°00.416 N, 111°24.563 W	4562; AT15-56 frame grabber coordinates	This publication
Notre Dame, **Figure 15**	27°N00.564 N, 111°24.410 W	4573; AT15-56 frame grabber coordinates	This publication

^∗^*The Guaymas samples in Ruff et al., 2015 are from Mat Mound (GB1, GB4a, GB4b), Megamat (GB2), and the UNC Mat (GB3).*

**FIGURE 1:**
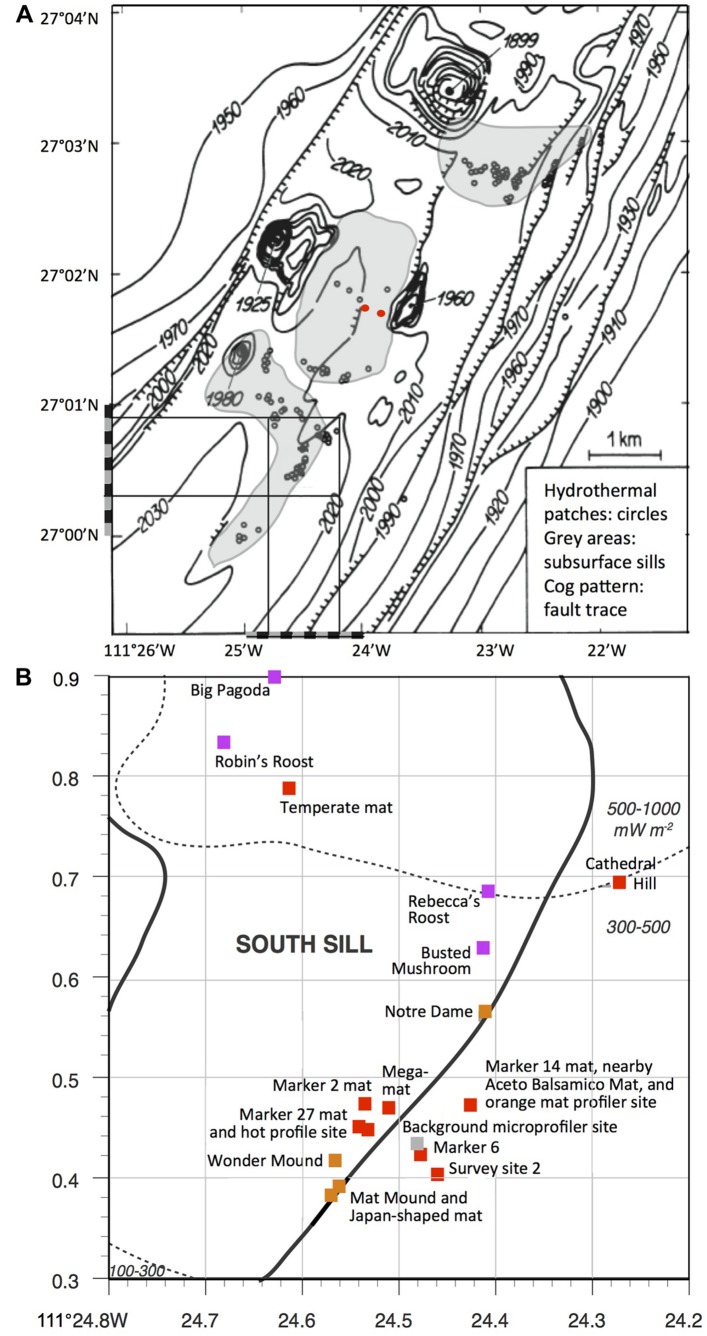
**Seafloor map of Guaymas Basin hydrothermal features.** **(A)** The southern Guaymas trough with hydrothermal sites marked as small circles (Bazylinski et al., 1989, adapted from Simoneit and Lonsdale, 1982). The square in the southwestern corner of the chart corresponds to the seafloor area shown enlarged and in greater detail below. The positions of the two DSDP drilling holes 477 and 477A are marked by red dots. **(B)** Sampling area of Guaymas cruises AT15-40 and 15-56, with major hydrothermal features, superimposed on heat flow measurements of Guaymas seafloor sediments (Fisher and Becker, 1991) and subsurface sill positions indicated by gray-shaded areas (Peter and Shanks, 1992). Red squares indicate mat-dominated sites, sienna brown indicates hydrothermal mounds with diffusive venting, and purple indicates large edifices and chimneys with evidence for channelized flow. The bathymetric map is reused from Bazylinski et al. (1989) and the sill positions are redrawn from Peter and Shanks (1992) by permission of the publishers.

We provide a baseline record of key locations and sampling sites that may be revisited on future research cruises, and be selected as targets for time-line studies of hydrothermal vent environments (**Figure 1**). We also place this frequently visited hydrothermal area into the context of underlying basalt sills emplaced into the sediment, and suggest that hydrothermal circulation patterns are not localized randomly; instead, this hydrothermal area and its hydrothermal flow paths follow specific boundaries of a shallow subsurface sill, or the fault lines through a sill (Lonsdale and Becker, 1985). This extended compilation may ultimately serve as a “hiking guide” that orients and familiarizes the reader and Guaymas Basin visitor with this uniquely complex seafloor landscape; like any guide, it will also lend itself to revisions, extensions, and updates that reflect ongoing and future research.

## Materials and Methods

### Image Localization and Retrieval

Images were recorded during research cruises with RV *Atlantis* and HOV *Alvin* during two cruises to the southern spreading center of Guaymas Basin in the Gulf of California (AT15-40, December 6–18, 2008, and AT15-56, November 23–December 5, 2009). Site locations were based on local XY grid data, the gridding system used by the National Deep Submergence Facility (NSDF) as recorded on the *Alvin* framegrabber system^1^ for the time and location of each observation. Local XY is a grid system (in meters) that is referenced to a local Latitude/Longitude origin. The conversion between Latitude/Longitude and Local XY use a simple flat-earth projection with East = X and North = Y. It is used for relatively small areas (a few kilometers) where the distortion introduced by the projection is minimal^2^. Latitude/longitude positions of key locations were converted from XY grid data using the online NSDF coordinate conversion utility^3^. To allow for unambiguous retrieval of the original images, each framegrabber image is referenced in the figure legends with the *Alvin* heading in which the image was taken (0 = north; 90 = east; 180 = south, 270 = west), the submersible’s depth in m, and the time point in Greenwich Mean Time (GMT). For higher-resolution images taken with *Alvin’s* external still camera, only GMT is given, as imprinted on the image file. When possible, photos were complemented with 10 cm scale bars calibrated by two red laser beams emitted from *Alvin*, 10 cm parallel from each other.

### Heat Flow Measurements

*In situ* temperature profiles at sampling sites were recorded using *Alvin*’s external heat flow temperature probe, a 0.6 m titanium tube containing a linear heater and five thermistors (type 44032, Omega Engineering, Inc.) at 10 cm intervals along the length of the tube (McKay et al., 2012). When fully inserted, this probe records the approach of probe temperatures to *in situ* temperatures at the sediment/water interface, and at 10, 20, 30, and 40 cm sediment depth. The probe was inserted for ca. 3–5 min during every measurement until the temperature readings stabilized.

### Microprofiler Deployments

High resolution depth profiles were measured with an *in situ* microprofiler unit. The unit has an electronic cylinder with the amplifiers for the microsensors (11 total) and a computer for data storage and motor control. On the bottom plate of the electronic cylinder microsensors for H_2_S, pH, O_2_, redox potential and temperature were mounted (Revsbech and Ward, 1983; Jeroschewski et al., 1996; De Beer et al., 1997). The cylinder can be moved vertically in steps down to 25 μm. The microprofiler was adjusted to a buoyancy of 18 kg in water. It was carried by *Alvin* to the selected sites and precisely positioned by *Alvin’s* hydraulic manipulator arm. The microprofiler was preprogrammed to measure profiles of 13 cm length with a step size of 250 μm. By gently pushing the profiler in the sediment, the sensor tips were adjusted to the starting position of ca. 3 cm above the sediment surface. A profile measurement was started by pushing the starter button with the arm of *Alvin*. Each profile measurement took ∼75 min, including a waiting time of 10 min to allow *Alvin* to leave the site and continue with other tasks. After completing a profile the sensors returned to the starting position and the unit was ready to be repositioned for new measurements. Results of *in situ* profiler measurements were compared to independent measurements of geochemical gradients, via porewater analysis, and to thermal profiles determined using the *Alvin* heat flow probe.

The pH and ORP microsensors were calibrated in standard buffers; the offset at the seafloor was obtained by comparing with values in a retrieved bottom water sample, obtained by *Alvin’s* Niskin bottles. The H_2_S microsensor was calibrated by adding 100-μL increments of a 500-mM Na_2_S stock solution to acidified seawater (pH < 3) at *in situ* temperature. Subsamples from the calibration solution were fixed immediately in 2% (wt/wt) zinc acetate, and the H_2_S concentration was determined spectrophotometrically with the methylene blue method (Cline, 1969). The *S*_tot_ profiles *in situ* were calculated from the H_2_S and pH profile using a pK_1_ of 6.64 (Jeroschewski et al., 1996). The O_2_ sensor was calibrated *in situ* using the signal in bottom water and in anoxic sediment (de Beer et al., 2006). The oxygen concentration in bottom water was determined from retrieved samples using Winkler titration (Hansen, 1999).

### Thermocouple Arrays

The thermocouple arrays consist of eight ∼50 cm long Ti-sheathed 1/8th inch O.D. type-J thermocouples that are mounted on an open frame made from titanium to prevent corrosion when exposed to the hot vent fluid. The sensing ends of the thermocouples within a cylindrical open frame are placed over the vent orifice while the other end of the thermocouples are connected to two sensor modules that contain electronics and remain at a safe distance from hot fluid (Pagé et al., 2008).

### Porewater Geochemical Analyses

Sulfate concentration measurements were completed shipboard; after centrifuging sediment-filled 15 ml tubes, the overlying porewater was filtered through 0.45 μm filters, acidified with 50 μl of 50% HCl and bubbled with nitrogen for 4 min to remove sulfide. Sulfate concentrations were then measured shipboard using a 2010i Dionex Ion Chromatograph (Sunnyvale, CA, USA) through Ag^+^ exchange columns (Dionex) to remove Cl^–^ (Martens et al., 1999). For sulfide, 1 ml porewater samples were combined with 0.1 M zinc acetate and concentrations were analyzed spectrophotometrically on the ship (Cline, 1969). Porewater concentrations of dissolved organic acids were measured via HPLC (Albert and Martens, 1997). Briefly, we used a Beckman Model 332 gradient liquid chromatograph in combination with an ISCO V4 UV/VIS detector and a Shimadzu CR3-A integrator. The detector had an IO-mm flow cell and was operated at 400-nm wavelength. The column used was a 22-cm Brownlee C8 cartridge with a 1.5 cm C8 guard column and either a 1.5-cm C8 or polymeric reversed-phase guard cartridge in the sample loop as a concentrator (Albert and Martens, 1997).

## Results

The results are structured into three distinct sections. The first section provides an overview on the wide range of microbial mats that thrive on hydrothermal sediments, and situates previous microbiology studies of specific mat locations by recovering the *in situ* context, complemented by porewater geochemical profiles of mat-covered sediments when available. The second section focuses on microsensor *in situ* measurements in microbial mats and seafloor sediments, and their microbiological and geochemical context. The third section summarizes field observations on hydrothermal mounds and chimneys.

### Microbial Mats on Hydrothermal Sediments

**FIGURE 2:**
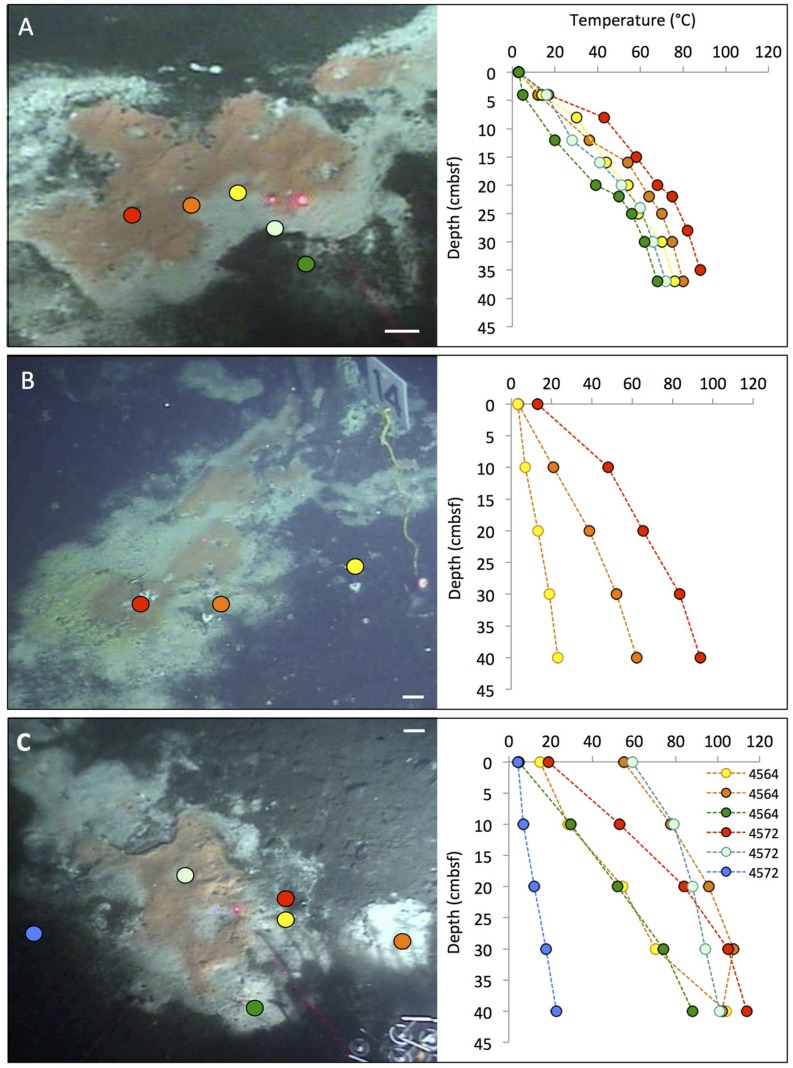
**Typical “fried egg” microbial mats.** The orange *Beggiatoa* mats in the center tend toward pink in these frame grabber images; yet direct *in situ* observation and shipboard recovery show orange as the actual color. *In situ* temperature gradients in the color-marked positions are measured with *Alvin’s* heat flow probe. The scale bar corresponds to 10 cm. **(A)** The Marker 2 mat was the first Guaymas Basin mat, where a temperature gradient from the center of the mat to the surrounding bare sediment was measured, using the *Alvin* high-temperature probe. *Alvin* heading 255, depth 2000.5 m, GMT 17:37:07, dive 4483, December 6, 2008. **(B)** The mat at Marker 14 was equipped with one temperature logger each in the orange center, the white fringe, and nearby bare sediment. *Alvin* heading 208, depth 2007.8 m, GMT 16:38:23, dive 4562, November 23, 2009. Geochemistry and temperature profiles of this mat are published (McKay et al., 2012); the temperature profiles are replotted here for comparison. **(C)** The large mat at Marker 27 is shown here before installment of temperature loggers or sampling. *Alvin* heading 36, depth 2002.5 m, GMT 22:02:30, dive 4564, November 25, 2009. Photographs courtesy of the Woods Hole Oceanographic Institution, from RV *Atlantis* cruises AT15-40 and AT 15-56.

Among the wide range of hydrothermal vent sites, Guaymas Basin is distinguished by hydrothermal sediments that are permeated by fluids rich in sulfidic, methane, and dissolved inorganic carbon (DIC; Von Damm et al., 1985). Where these fluids reach the sediment surface, they sustain highly visible microbial mats of large, filamentous, vacuolated, sulfur-oxidizing bacteria within the family *Beggiatoaceae* (Jannasch et al., 1989; Nelson et al., 1989). These microbial mats often show a white fringe and an orange center, reminiscent of fried eggs, and contrast sharply against the surrounding brown–gray sediment (**Figure 2**). The orange center coincides with local maxima of temperature, carbon and energy sources; upward-shifted temperature zones and concentration peaks of hydrothermal energy sources characterize the underlying sediments. The white filaments are sustained by more gradual hydrothermal gradients on the periphery of the hydrothermal hot spot. Interestingly, noticeable temperature and chemical gradients extend into the surrounding bare sediments, but they become less steep and do not sustain thick mats (Supplementary Figure S1, and McKay et al., 2012). In these cases, essential components for seafloor mat sustenance appear to be lacking, or are not sufficient for colonization by mat-forming bacteria. The orange-colored and colorless filaments were placed near the genus-level candidate taxa Maribeggiatoa and Marithioploca, and the genus *Thiomargarita* based on 16S rRNA sequencing of individual filaments (McKay et al., 2012); the common literature designation of these organisms as *Beggiatoa* (Jannasch et al., 1989; Nelson et al., 1989) should be regarded as shorthand for what are actually several distinct genus-level lineages within the family *Beggiatoaceae* (Salman et al., 2011; Teske and Salman, 2015). Genome- and protein-based studies of the orange filament type indicate that they are versatile organisms with autotrophic and heterotrophic capabilities that can oxidize sulfide with nitrate as electron acceptor (MacGregor et al., 2013a,b). At a sediment depth of a few centimeters below these mats, thermotolerant, anaerobic methane-oxidizing archaea (ANME archaea) are frequently detected in 16S rRNA gene clone libraries and high-throughout sequencing surveys (Teske et al., 2002; Biddle et al., 2012; Ruff et al., 2015; Dowell et al., 2016). The ANME archaea show distribution patterns that are congruent with the high concentration of methane (several millimolar) in the hydrothermal sediments; they are in part represented by high-temperature adapted lineages in Guaymas Basin (Holler et al., 2011; Biddle et al., 2012; Kellermann et al., 2012). The combination of ANME archaea in anaerobic, reducing and methane-rich sediments, and of sulfide-oxidizing *Beggiatoa* mats or other sulfide-oxidizing bacteria on the surface of the same sediments, is highly characteristic of Guaymas Basin hydrothermal sediments (Teske et al., 2014).

The mat shown in **Figure 2C** was sampled during dive 4572 for a biogeochemical study of nitrate reduction in hydrothermal sediments of Guaymas Basin. Surficial (0–3 cm) sediments of four *Alvin* push cores in the white mat between the yellow and green heat flow gradients of Dive 4564 (near core 4564-1, plotted in Supplementary Figure S1) were used to test the response of nitrate reduction to increased concentrations of nitrate (positive at 0.5 mM nitrate and higher), sulfide (negative at >0.5 mM sulfide), and DOC (no effect from 0 to 5 mM DOC carbon; Bowles et al., 2012). Therefore, high sulfide concentrations interfere with nitrate reduction in the hydrothermal sediments of Guaymas Basin, just as previously observed in coastal and estuarine sediments (Joye, 2002). Interestingly, a parallel 16S rRNA gene sequencing survey of these Guaymas mat sediments did not yield any members of the *Beggiatoaceae*, but mostly members of Delta- and Alphaproteobacteria, the Bacteroidetes, and some Gammaproteobacteria (Bowles et al., 2012). Due to their high cell volume, the *Beggiatoaceae* contribution to cell numbers and genomes in microbial mats lags behind that of other abundant bacteria, and they easily elude sequence-based detection; capturing their 16S rRNA genes requires highly purified filaments (Jørgensen et al., 2010; McKay et al., 2012). Taken together, these studies indicate that by cell and genome number per volume, members of the *Beggiatoaceae* may not even be the most abundant bacteria that perform nitrate reduction in organic-rich, sulfide-rich mat sediments of Guaymas Basin mats and hydrothermal sediments; nitrate-reducing, sulfide- or hydrogen-oxidizing Epsilonproteobacteria are plausible candidates for this ecological role in hydrothermal environments (Campbell et al., 2006).

**FIGURE 3:**
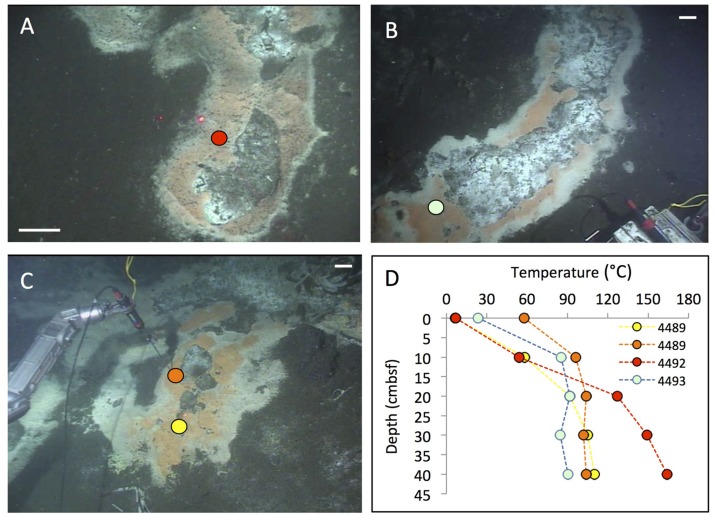
**Cratered microbial mats.** Microbial mats with a white outer fringe, an orange layer, and a “cratered” central area with white-coated sediments, coinciding with shimmering water. *In situ* temperature gradients are measured with the *Alvin* heat flow probe. The scale bar corresponds to 10 cm. **(A)** Small elongated mat connected to a small mound outside of the top picture frame. Two cores have been sampled here under the moniker “Survey site 2” (Meyer et al., 2013). *Alvin* heading 334, depth 2011 m, GMT 19:58:15, dive 4492, December 16, 2008. **(B)** Elongated “Japan-shaped” mat was connected to a small mound outside of the upper picture frame. *Alvin* heading 237, depth 2004 m, GMT 20:13:25, dive 4493, December 17, 2008. Examination of the framegrabber record showed that this mat was also observed just before sighting Mat Mound (**Figure 13**) at GMT 18:34:40, with a heading of 306 on dive 4483. **(C)** The UNC mat, ca. 20 m southwest of Megamat (Biddle et al., 2012), contains a small cratered area in the center. *Alvin* heading 106, depth 2001 m, GMT 21:36:50, dive 4489, December 13, 2008. **(D)** The four temperature gradients from these cratered mats, plotted together. Photographs courtesy of the Woods Hole Oceanographic Institution, from RV *Atlantis* cruise AT 15-40.

In some cases, the fried-egg appearance of a hydrothermal hot spot is modified when the central orange mat turns into an orange fringe mat surrounding a central crater-like region, where the smooth mat surface gives way to roughly textured sediment, often with a dusting of white particles, probably sulfur precipitates (**Figure 3**). In some cases, shimmering water could be observed rising from the exposed, cratered sediment surface, indicating that the hydrothermal temperature gradient has an advective component. *In situ* microsensor studies of thick mats have shown hydrothermal circulation patterns that alternate between injections of oxygenated seawater and ejections of anoxic hydrothermal fluid through the sediment–water interface (Gundersen et al., 1992). Episodic intensification of such pumping patterns could disrupt and sweep away a microbial mat and surficial sediment, or exterminate the bacterial mat by sudden upflow of hot hydrothermal fluid or lack of a consistent redox gradient. While Guaymas Basin *Beggiatoaceae* depend upon the hydrothermal hot spot as a source of electron donors, they appear to prefer cool *in situ* temperatures (on average 8 to 12°C) at the sediment surface (McKay et al., 2012).

**FIGURE 4:**
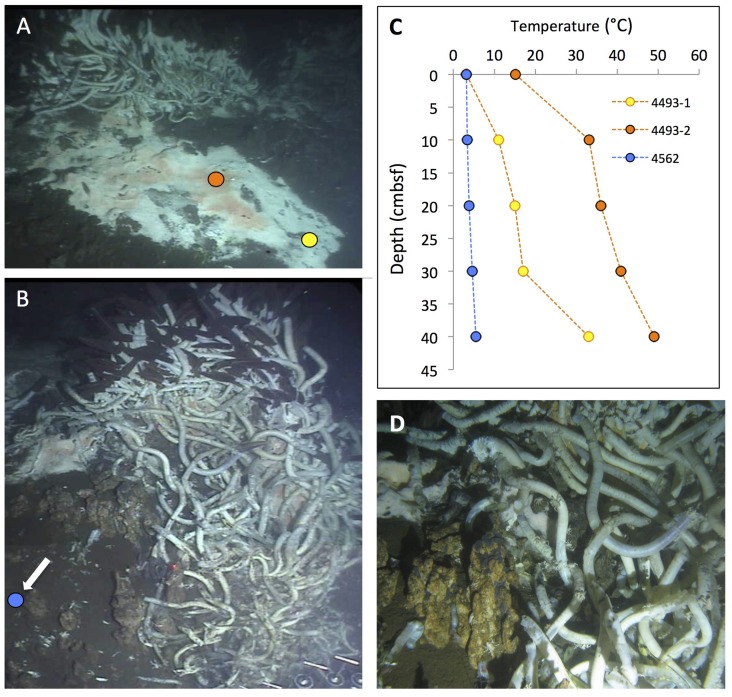
**Changing hydrothermal flux.** The changing microbial mats and *Riftia* colonies at this small hydrothermal mound, termed “Marker 6,” were documented during repeat visits in December 2008 and November 2009. **(A)** Framegrabber image of active *Beggiatoa* mat on sloping sediment next to thriving *Riftia* cluster on small mound. Two heat flow probe measurements revealed hydrothermal gradients in the white and orange mat. *Alvin* heading 123, depth 2003 m, GMT 22:19:44, dive 4484, December 7, 2008. **(B)** Composite framegrabber image of the same spot at a clockwise rotated angle, taken almost a year later. A cold heat flow profile (see arrow on left margin) measured at a spot between the two hot 2008 temperature profiles shows that the hydrothermal temperature gradient has almost disappeared. The *Beggiatoa* mat has receded to a small spot adjacent toward and into the center of the *Riftia* cluster; the sedimented slope is bare and much of the *Riftia* cluster has died, leaving empty sheaths dominated by scavenging isopods in the foreground. *Alvin* heading 91, depth 2006 m, GMT 17:47:25 (lower portion) and 17:47:55 (upper portion of composite image), Dive 4562, November 23, 2009. **(C)** Temperature gradient plots of the two active 2008 gradients and the cold 2009 gradient. **(D)** Closeup of decaying *Riftia* with abundant crabs and scale worms in the foreground of **(B)**; the image was taken with the external *Alvin* still camera. GMT 17:22:57. *Alvin* Dive 4562, November 23, 2009. Photographs courtesy of the Woods Hole Oceanographic Institution, from RV *Atlantis* cruise AT 15-56.

Although hydrothermal hot spots with *Beggiatoa* mats are highly conspicuous, they are transient seafloor features that depend on the changeable course of hydrothermal flow paths in the subsurface. For example, a well-developed white and orange *Beggiatoa* mat overlying hydrothermally active sediment next to a healthy *Riftia* colony on a small hydrothermal mound (**Figure 4A**), visited in December 2008 and identified by placing a marker disk nearby, had almost entirely disappeared a year later (**Figure 4B**), along with the hydrothermal temperature gradient in the sediment (**Figure 4C**). The *Riftia* colony was decaying on its periphery, and scavengers (scale worms and isopods) occurred in conspicuous abundance (**Figure 4D**). The rusty-brown color of the hydrothermal outcrops, especially visible in **Figure 4D**, indicate oxidizing conditions, the absence of microbial mat overgrowth, and the scarcity or absence of dissolved sulfide; such conditions are incompatible with active *Riftia* sp. (Luther et al., 2001), and have instead been documented as the declining stage of *Riftia* colonies (Shank et al., 1998).

**FIGURE 5:**
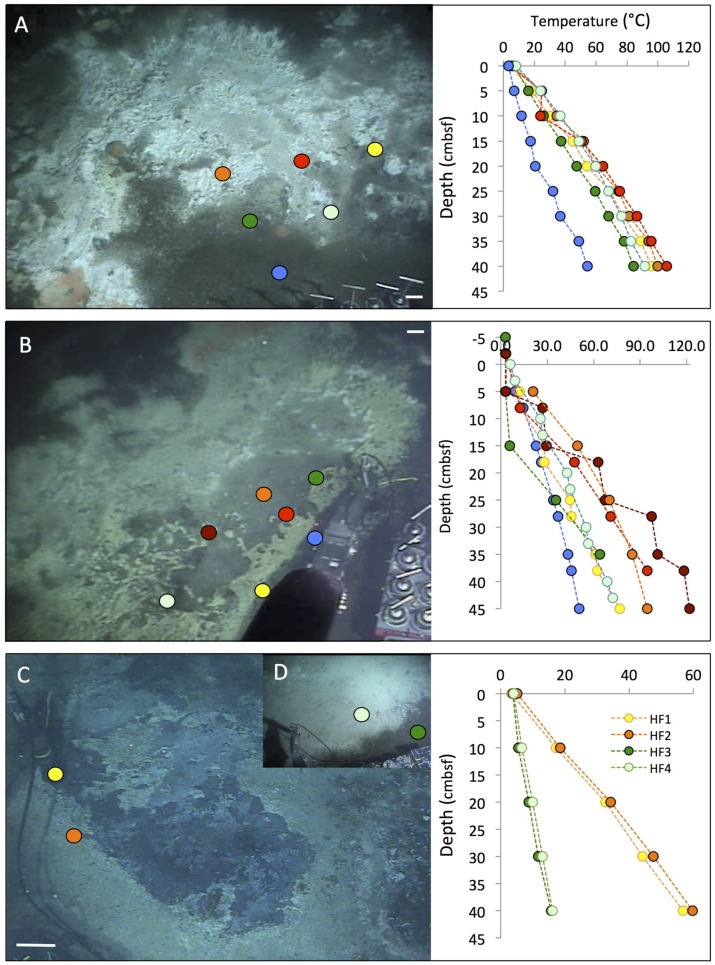
**Megamat and Cathedral Hill mats.** Temperature profiles measured with the *Alvin* heat flow probe are marked with colored dots on the photos and plotted profiles in matching colors. The scale bar corresponds to 10 cm. **(A)** Partial view of hydrocarbon-rich Megamat before coring and sampling. The heat flow profiles show the thermal transition from mat-covered to bare sediment. Alvin *heading* 219, depth 2012 m, GMT 18:14:26, dive 4562, November 23, 2009. **(B)** Overview of the microbial mats area at the Cathedral Hill area, a complex of multiple white, gray, yellow, and orange mats next to a small hydrothermal massif. *Alvin* heading 174, depth 2020.3 m, GMT 18:33:10, dive 4565, November 26, 2009. Seven heat flow temperature gradients were measured on *Alvin* dive 4565 during a general survey of the area. **(C)** Temperate mats resembling the Cathedral Hill mats by yellow, white and gray surface colors. *Alvin* heading 126, depth 2021 m, GMT 18:37:07, dive 4574, December 5, 2009. **(D)** The small insert in the upper right corner shows a nearby, significantly cooler, white–gray and smooth mat area. *Alvin* heading 120, depth 2020.7 m, GMT 19:17:40, dive 4574, December 5, 2009. Photographs courtesy of the Woods Hole Oceanographic Institution, from RV *Atlantis* cruise AT 15-56.

In addition to the commonly encountered *Beggiatoa* mats associated with hotspots of reducing hydrothermal fluids migrating toward the sediment surface, several previously unreported types of mats and colorful surface precipitates have been found that remain to be investigated more closely (**Figure 5**). An unusually extensive hot mat was called “Megamat” for its size as well as its extremely hot temperatures, and repeatedly sampled in 2008 and 2009 (Biddle et al., 2012; Cardman, 2014). Most of this mat was covered with white precipitates; some marginal areas contained yellow and orange *Beggiatoa* mats (**Figure 5A**). The sediments of Megamat contained smaller proportions of methane-oxidizing archaea (ANME), but yielded abundant phylotypes of hyperthermophilic archaea, including the acidophilic, sulfur-reducing archaeon *Aciduliprofundum thermophilum* (Cardman, 2014). Based on multiple subsurface temperature gradients in the hottest region of Megamat, the temperature field within the underlying sediments was modeled in 3D, and showed temperature gradients steepening from the margins of the mat toward its central region, where 200°C was recorded at ca. 35 to 40 cm depth (McKay et al., 2012). Steep temperature gradients across the edge of Megamat were also found in 2009; within ca. 1 m distance, the temperature gradient of ca. 50°C over 40 cm depth in the bare sediment area adjacent to the mat doubles to ∼100°C over the same depth (**Figure 5A**). The porewater profiles of Megamat indicate methane- and DIC-rich sediments where sulfate decreases toward depletion and sulfide accumulates in the upper sediments layers (Supplementary Figure S2).

A distinct mat complex near a group of small hydrothermal mounds and chimneys, called “Cathedral Hill,” consisted of a complex mosaic of grayish-colored sediments partially covered by yellow precipitates (**Figure 5B**). This area was extensively mapped with seven temperature profiles and turned out to be consistently warm or hot, as the sediment gradients varied from ca. 50°C to 120°C at 40 cm depth (**Figure 5B**); matching porewater profiles indicated methane- and DIC-rich, highly sulfidic sediments with rapid sulfate depletion below the sediment surface (Supplementary Figure S3). The conspicuous yellow precipitates on the sediment surface are not *Beggiatoa* mats; microscopic examination of such a sediment core (core 4565-6, next to the thermal profile in yellow) revealed filamentous sulfur precipitates that are commonly produced by autotrophic sulfide-oxidizing bacteria of the genus *Arcobacter*, a member of the Epsilonproteobacteria (Pjevac et al., 2014). Since sulfur precipitates produced by *Arcobacter* sp. are usually bright white (Taylor et al., 1999), additional factors would be required to account for the yellowish color; a close investigation of these precipitates is certainly warranted.

Cooler sediments harbored similar, complex yellow-tinted or light-gray precipitates surrounded by brown seafloor sediments. These mats appeared as a thick but uneven carpet of precipitates, with an interconnected web of “ridges” and lower lying portions between these ridges (**Figure 5C**).

### *In Situ* Microprofiler Measurements

**FIGURE 6:**
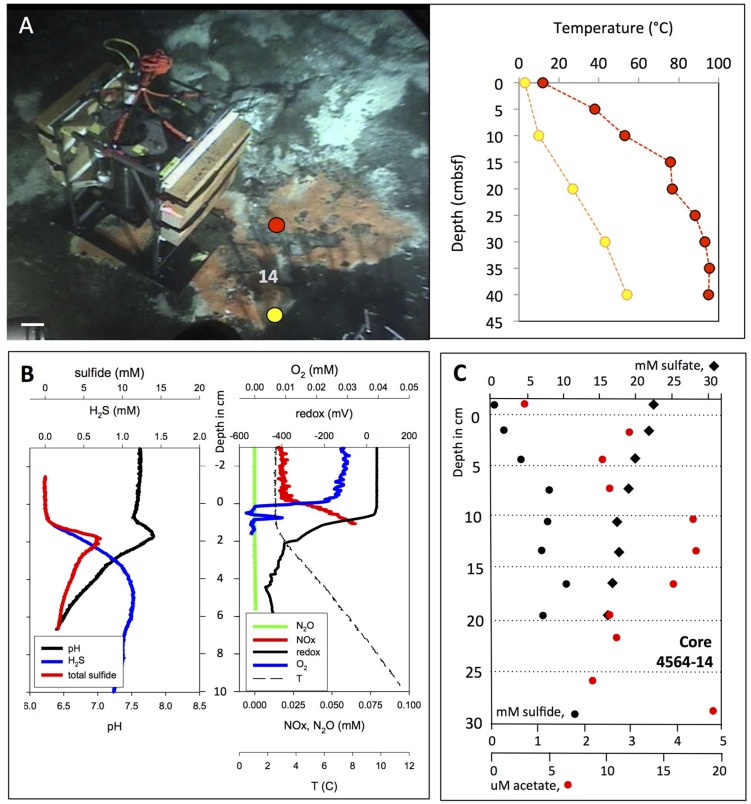
***In situ* microprofiler survey of orange *Beggiatoa* mat.** **(A)** *In situ* profiler after placement on an orange *Beggiatoa* mat near Marker 14. *Alvin* heading 96, depth 2008 m, GMT 16:36:50, dive 4564, November 25, 2009. Temperature gradients were measured with the heat flow probe in two spots near the base of the profiler. The microsensor temperature profile matches the near-surface heat flow temperature profile plotted in yellow, but it is cooler than the nearby heat flow profile in red. **(B)** *In situ* profiler measurement of temperature, redox potential, oxygen, nitrate/nitrite, N_2_O, H_2_S, and total sulfides at orange mat location. **(C)** Sulfate, sulfide, and acetate porewater gradients from *Alvin* core 4564-14 taken in orange mat-covered sediment, marked by the number 14 in panel **(A)**. Photographs courtesy of the Woods Hole Oceanographic Institution, from RV *Atlantis* cruise AT 15-56.

**FIGURE 7:**
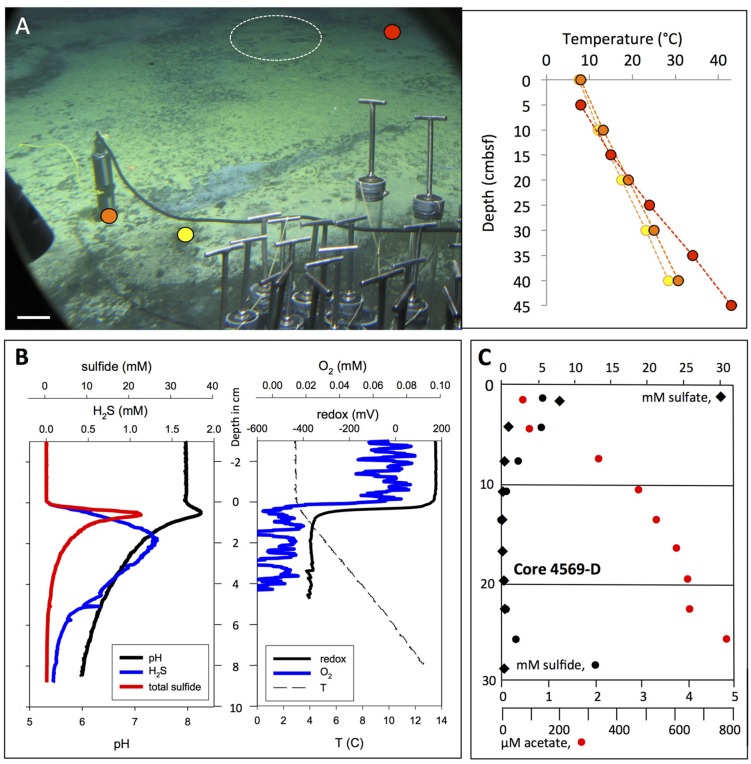
***In situ* microprofiler survey of yellow “Aceto balsamico” mat.** **(A)** This mat on moderately warm sediments resembles the yellow, sulfur-rich Cathedral Hill mats in sulfur color and surface texture; it is located ca. 10 m south of Marker 14. The scale bar corresponds to 10 cm. Corresponding frame grabber images: *Alvin* heading 218, 2009.5 m depth, GMT 16:59:05, dive 4562, November 23, 2009. Two heat flow probe measurements were made on dive 4562, marked in yellow and orange. The *in situ* microprofiler measurement was performed in the area marked by the circle during *Alvin* dive 4570 (November 24, 2009), and a heat flow measurement nearby (in red) was taken. **(B)** *In situ* microprofiler measurement of temperature, redox potential, oxygen, nitrate/nitrite, N_2_O, H_2_S, and total sulfides during *Alvin* dive 4570. **(C)** The sulfide, sulfate and acetate porewater profiles are from core D taken on dive 4569. Photographs courtesy of the Woods Hole Oceanographic Institution, from RV *Atlantis* cruise AT 15-56.

**FIGURE 8:**
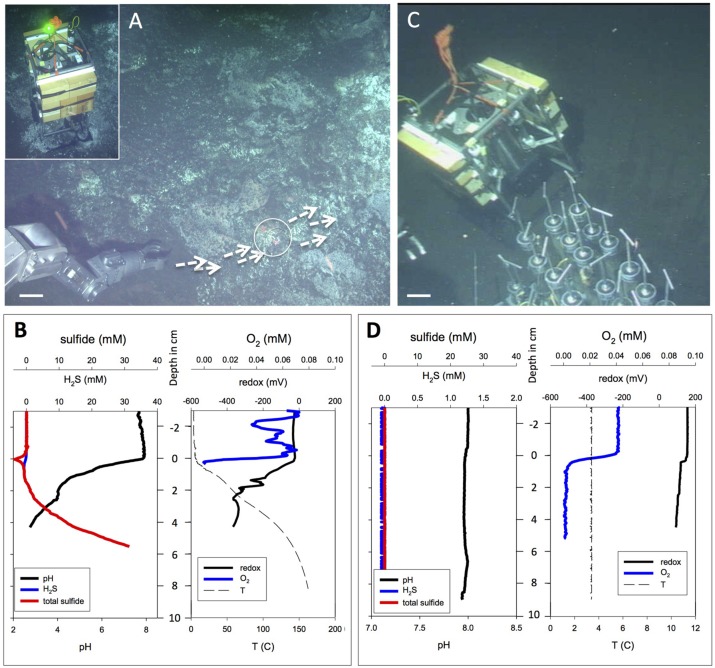
**Extremely hot versus background microprofiler deployments.** Contrasting extremely hot and non-hydrothermal background microprofiler deployments. **(A)** *In situ* microprofiler deployment near Marker 27, on sediment with strong hydrothermal flow and schlieren patterns along the sediment–water interface. White arrows mark surface fluid flow; the circle marks the approximate area of the microprofiler measurement. The scale bar corresponds to 10 cm. *Alvin* heading 287, depth 2001 m; GMT is unknown due to *Alvin* frame grabber gap; dive 4566, November 27, 2009. The insert in the upper left corner shows the microprofiler just as it initiates the measurements at the hot spot, as indicated by a green light signal. The frame grabber record documents this profiler location again on the subsequent day: *Alvin* heading 160, depth 2001 m, GMT 20:59.32, dive 4567, November 28, 2009. **(B)** *In situ* profiler measurement of temperature, redox potential, oxygen, H_2_S, and total sulfides at the hot flow spot during *Alvin* dive 4566. **(C)** *In situ* microprofiler deployment on bare olive-brown sediment without visible hydrothermal activity or microbial mats. The scale bar corresponds to 10 cm. *Alvin* heading 216, depth 2001 m, GMT 19:14:13, dive 4569, November 30, 2009. This deployment provides a negative control. A heat flow gradient taken next to the profiler showed cold seafloor temperatures between 2.8 and 3.0°C throughout its depth range. **(D)** *In situ* profiler measurement of temperature, redox potential, oxygen, H_2_S, and total sulfides on cold, non-hydrothermal sediments during *Alvin* dive 4569. Photographs courtesy of Woods Hole Oceanographic Institution, from RV *Atlantis* cruise AT 15-56.

Several mats were examined by *in situ* microprofiling, to obtain finely resolved thermal and chemical gradients across the mat surface on the millimeter and centimeter scale; the profiles shown here cover a vertical extent of 10 cm. We examined orange *Beggiatoa* mats overlying hydrothermally active sediments (**Figure 6**), yellow mats or precipitates overlying relatively cool sediments (**Figure 7**), sediments with advective flow of extremely hot hydrothermal fluid (**Figures 8A,B**), and cold background sediments without visible hydrothermal activity (**Figures 8C,D**). Shared features of these microprofiler measurements were the low oxygen concentrations recorded above the sediment surface, ranging from near 30 to ca. 60 μM, or ca. 10 to 20% of seawater saturation. These values, measured in cold bottom water above the sediment surface, are unlikely to represent measurement artifacts caused by hydrothermal impact on the probes, since the control measurements above cold, non-hydrothermal sediment also yielded bottom water oxygen concentrations near 40 μM, in the same range as above hydrothermal sediments (**Figure 8D**). Oxygen concentration profiles of the Guaymas Basin water column obtained over 50 years ago are consistent with the microsensor data and indicate strong oxygen depletion throughout the mid- and bottom water, toward ca. 20% of near-surface oxygen concentrations (Calvert, 1964).

The orange *Beggiatoa* mat (**Figure 6A**) and some of its associated microprofiler gradients (total sulfide, O_2_ and NO_x_) were previously published as “*Beggiatoa* Mat BM1” (Winkel et al., 2014), but are documented here in full. The local thermal gradients at the microprofiler site showed considerable spatial heterogeneity (**Figure 6B**). While the heat flow temperature gradient in yellow (**Figure 6A**) and the *in situ* temperature gradient determined by the *in situ* profiler (**Figure 6B**) resembled each other and showed a temperature increase of ∼1°C per cm almost linearly over the measurement range, the heat flow temperature gradient in red (**Figure 6A**) was considerably steeper and reaches higher temperatures, illustrating the high degree of spatial heterogeneity.

The orange *Beggiatoa* mat shows sharp chemical changes at the mat surface (**Figure 6B**). The concentrations of H_2_S and total sulfide, which are not detectable in the bottom water and at the mat surface, increase sharply downcore. H_2_S concentrations rose along a strong linear gradient within the upper sediment layers until they reached a concentration plateau of ca. 1 mM near 4 cm depth, indicating no net production or consumption. Total sulfide accumulated to a local maximum around 5 mM in the upper 2 cm before slowly decreasing (**Figure 6B**). The total sulfide peak indicates desulfurization reactions in the sediment, mobilization and upward migration of reduced sulfur, and precipitation and accumulation of these sulfur phases immediately below the sediment surface. The shape of the total sulfide profile, below the surficial total sulfide peak, indicated fluid upflow of ca. 50 cm/year (de Beer et al., 2006). The pH values showed a surface-associated maximum near pH 8, consistent with several possible explanations. Chemosynthetic activity and CO_2_ uptake into microbial cells could deplete CO_2_ locally, analogous to photosynthetic CO_2_ depletion in benthic cyanobacterial mats (Jørgensen et al., 1983); however, chemosynthetic sulfur oxidation and its resulting acidification effects argue against this explanation. Electron transport and H^+^ consumption by cable bacteria could account for this slightly alkaline peak at the sediment surface (Nielsen et al., 2010). Another contributing reaction for this pH could be the proton-consuming microbial oxidation of H_2_S with nitrate to elemental sulfur and dinitrogen gas or ammonia; the reactants are available in the surficial sediment (Salman et al., 2015). The pH then decreased to near-neutral levels around 6.5 downcore, approaching the mildly acidic pH of 5.9 for carbonate-buffered hydrothermal fluids measured *ex situ* at Guaymas Basin (Von Damm et al., 1985). Combined nitrate and nitrite concentrations increased from bottom water background of ∼20 μM (most likely dominated by nitrate) to ca. 75 μM within the mat, possibly indicating intracellular nitrate accumulation and leakage by large, vacuolated *Beggiatoaceae* (McKay et al., 2012), and nitrifying activity by ammonia-oxidizing, nitrite-producing archaea that grow associated with the *Beggiatoaceae* filaments (Winkel et al., 2014). Oxygen was quickly consumed at the mat surface; a narrow local peak within the upper 1 cm of the mat may indicate advective transport, for example by hydrothermal pumping that re-introduces pockets of oxygenated seawater into shallow sediments near hydrothermal hot spots (Gundersen et al., 1992). Regardless of short-term oxygen spikes, the redox potential of the upper sediment decreased below 400 mV in the upper 2 cm, indicating consistently reduced conditions.

The sulfide profile determined by *in situ* profiler was consistent with the porewater profile of H_2_S measured in core 4564-14 next to the *in situ* profiler; porewater sulfide reaches the 1 mM range between the midpoints of the 3–6 and 6–9 cm sediment layers, and remains generally between 1 and 1.5 mM throughout the remaining length of the core (**Figure 6C**). The lack of fine-scale resolution and the slightly lower H_2_S porewater concentrations in the surficial sediments are consequences of porewater processing and potential sulfide loss due to oxidation.

An extensive yellow mat found during dive 4562 was characterized by similar yellow-colored surface precipitates as seen at Cathedral Hill, but showed moderate temperature gradients reaching 30°C (**Figure 7A**). This mat was investigated by microprofiler deployment (**Figure 7B**) and push coring followed by porewater analysis (**Figure 7C**). The geochemical and temperature gradients in this mat differed from those in the orange *Beggiatoaceae* mat examined on dive 4564. The *in situ* temperature microprofile started with the bottom water temperature (ca. 3.5°C, **Figure 7B**) at the sediment surface, whereas the heat flow probes started with ∼5°C higher temperatures (**Figure 7A**). This is a possible consequence of inserting the relatively thick heat flow probe into the sediment as it creates a flow channel during insertion into the sediment. After detecting high acetate porewater concentrations in its underlying sediment, represented here by an acetate porewater profile from core 4569-D (**Figure 7C**), the mat was nicknamed “Aceto Balsamico Mat.” The geochemical gradients in this mat differ from those in the orange *Beggiatoaceae* mat examined on dive 4564. The porewater acetate concentrations reaching >800 μM in the mat subsurface sediments exceeded the moderate acetate concentrations – in the range of 10–20 μM – that were found in the *Beggiatoa* mat sediments examined during dive 4564 (**Figure 6C**). The lower H_2_S concentrations in the mat measured in core 4569-D were confirmed independently by *in situ* profiling during dive 4570. H_2_S concentrations decreased from the 1 mM range at the sediment surface to detection background below 10 cm sediment depth. Total sulfide at the sediment/water interface reached 20 mM (**Figure 7B**). As porewater sulfide disappeared within the upper 10 cm, porewater sulfate was depleted toward background within the upper 10 cm (**Figure 7C**). This simultaneous disappearance of sulfate and sulfide is highly unusual among all Guaymas Basin sediment profiles; its explanation would call either for a sulfur-depleted subsurface fluid source, or incomplete sulfate reduction or sulfide oxidation to intermediate oxidation states of sulfur. The noisy oxygen profile (**Figure 7B**) was characterized by omnipresent irregular oscillations between individual measurement points, spaced by 250 μm, above and within the sediment. If taken literally, these oscillations would indicate strongly fluctuating oxygen concentrations on submillimeter vertical scales within the bottom water, which seems unlikely; instead, unidentified *in situ* conditions might have interfered with the stability of the oxygen probe readings. The oscillations were superimposed on a pattern of rapid oxygen consumption at the mat surface, a possible consequence of sulfide oxidation at the sediment surface. The redox profile remained smooth and changes from close to +200 mV in the bottom water to ∼–400 mV in the sediment (**Figure 7B**).

A microprofiler deployment into very hot sediment (**Figures 8A,B**) during Dive 4566 has to be regarded as exploratory, since the microelectrodes have not been tested and evaluated for extremely high temperatures. The sediment was covered by irregular white patches and crusts that do not resemble the thick white and orange *Beggiatoaceae* mats. Instead of gradual, diffusive hydrothermal seepage, advection seems to play a greater role here; swirl-like optical distortions or “schlieren patterns” in the bottom water rippling over the sediment surface (indicated by arrows in **Figure 8A**) indicate warm or hot fluid flow directly on the sediment surface and across the spot where these microprofiles were measured, marked by a circle. The shimmering effect can indicate escaping hydrothermal fluids, or convective heating from subsurface fluid conduits that cause convection from the heated sediment surface to the cold seawater. Sulfide concentrations reached 30 mM within 6 cm of the sediment surface; these extremely high sulfide concentrations require a strong hydrothermal contribution. Total sulfide and H_2_S concentrations are expected to be identical, as the pH is far below the pK_1_ value for H_2_S/HS^–^. Oxygen fluctuations in the overlying water could be the equivalent of centimeter-scale perturbations of oxygen-depleted water rising from and moving along the sediment surface (**Figure 8B**). The oxygen probe did not work within the hot sediment. The extreme temperature gradient reaches ca. 160°C at 8 cm depth; and the associated pH gradient converges to at least pH 2.5 below 4 cm depth. Such pH extremes require the presence of strong acids under minimal alkalinity and low DIC, conditions that are at odds with the reported pH of 5.9 for carbonate-buffered Guaymas Basin hydrothermal fluid (Von Damm et al., 1985). If correct, this measurement would indicate the presence of unbuffered, strongly acidified hydrothermal fluids. It is also possible that the microsensor technology used here is running against its inherent limits at high *in situ* temperatures, and that alternative sensor materials, for example pressure- and temperature-stable iridium oxide sensors, are required (Kakooei et al., 2013).

Cold Guaymas Basin seafloor sediments without any visible mat cover were profiled as a negative control (**Figure 8C**); they showed the absence of sulfide, a consistent porewater pH near 8.0 (close to seawater pH), non-reducing conditions, and uniformly cold temperatures near 3.5°C in the sediment (**Figure 8D**). Under these cold, non-hydrothermal conditions, the microsensor signals remain smooth and do not show any unusual distortions or oscillations.

### Hydrothermal Chimneys and Mounds

The hydrothermal edifices in Guaymas Basin show diverse morphologies that can be categorized into broad mounds, thick chimneys, and thin flutes and flanges; their complex composition represents a mixture of carbonates, sulfates, silicates, metal sulfides, and iron oxides, and further distinguishes them from the metal sulfide-dominated deposits and chimneys at sediment-free spreading centers (Koski et al., 1985; Lonsdale and Becker, 1985; Peter and Scott, 1988). Broad hydrothermal mounds with extensive talus slopes do not show conspicuous venting, but their surfaces, often sealed by amorphous silica precipitates, cover internal hydrothermal circulation. Hydrothermal chimneys with thick trunks of cemented hydrothermal sulfides and carbonates are often locally overgrown with microbial mats and *Riftia* clusters under suitable diffusive venting regimes. Extremely hot, fragile and highly active venting structures composed of hydrothermal sulfide minerals can take the shape of thin, vertically growing flutes or horizontally spreading eaves and flanges (Peter and Scott, 1988). These flutes and flanges often appear at the top or on the flanks of larger hydrothermal edifices where they mark locations of channelized hydrothermal outflow; generally they are too hot to allow growth of microbial mats.

**FIGURE 9:**
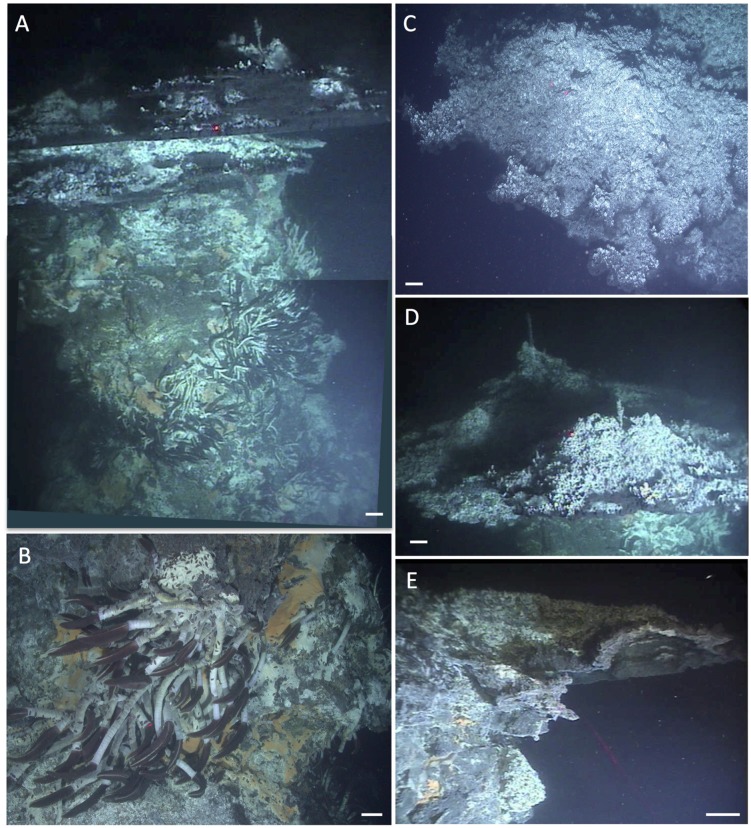
**Hydrothermal flanges at Big Pagoda.** This large hydrothermal edifice, visited during dive 4574 on December 5, 2009, is termed “Big Pagoda” in reference to its flanges that are spreading like protruding pagoda roofs at the top of this structure. Scale bars correspond to 10 cm. **(A)** Composite image of Pagoda showing the flanges emerging from the top of the massive hydrothermal edifice, its trunk overgrown with microbial mats and *Riftia* clusters. The top section of this image has heading 172; depth 1979.7 m, GMT 16:37:05, and bottom image section has *Alvin* heading 179, depth 1980 m, GMT 16:36:35. **(B)** Close-up of faunal assemblage on Pagoda trunk, with *Riftia* clusters, orange and white *Beggiatoa* mats, and a profusion of grazing scale worms. GMT 16:44:43. **(C)** Top-down view of spreading flange, GMT 16:37:36. Photos **(B,C)** were taken with *Alvin’s* still photo camera. **(D)** Side view of Pagoda top, with the same flange in the foreground. *Alvin* heading 156, depth 1979 m, GMT 16:38:05. **(E)** View of protruding flange section from below, as it emerges from the base of the hydrothermal edifice on the left. *Alvin* heading 23, depth 1980.3 m, GMT 16:43:05. Photographs courtesy of Woods Hole Oceanographic Institution, from RV *Atlantis* cruise AT 15-56.

A good example for a large hydrothermal edifice that combines a thick trunk with flanges on top is the “Big Pagoda” structure (**Figure 9**). Shown here is a section of ca. 3.5 m in vertical extent with microbial mats and *Riftia* colonies growing on the trunk, indicating diffuse venting (**Figures 9A,B**). The top of the edifice is covered with lobed extensions spreading approximately a meter sideways into the water column (**Figures 9C–E**). Broken-off inactive flanges appeared underneath larger, more recent flanges, indicating their continuous formation and extension concomitant with degradation and erosion (**Figure 9E**). Small chimneys appeared in the center of some flanges (**Figures 9A,D**), indicating that part of the hydrothermal flow seeps and rises through the center of these protuberances. The orange and white *Beggiatoa* mats that are abundant on the central trunk of “Pagoda” were missing on the flanges.

**FIGURE 10:**
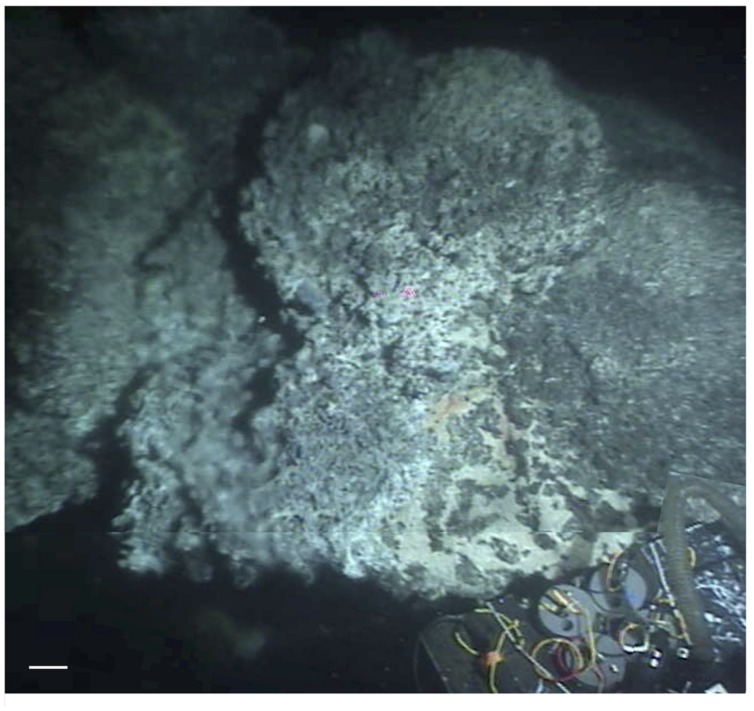
**Robin’s Roost.** This hydrothermal structure was visited during dive 4574 on December 5, 2009. Its top shows actively venting flanges, partially overgrown with *Beggiatoa* mats. Using *Alvin’s* high-temperature probe, an *in situ* temperature of 278.5°C was measured in the shimmering vent fluids rising at and around the lip of the flange, on the left within this composite frame grabber image. Yellow and orange *Beggiatoa* mats are growing in protected spots on top of the flange, in the right center of the composite image. The sampling box in the lower right is ca. 50 cm wide. The scale bar corresponds to 10 cm. *Alvin* heading 151, depth 2000.5 m, GMT 18:18:07, and 18:17:07 for the bottom part of the image. Photographs courtesy of Woods Hole Oceanographic Institution, from RV *Atlantis* cruise AT 15-56.

A smaller hydrothermal site termed “Robin’s Roost” showed the highly localized hot venting area underneath an active flange, where an *in situ* temperature of 278.5°C was measured in shimmering vent fluids rising over its outer edge (**Figure 10**), using *Alvin’s* high-temperature probe. The portion of the flange that is not directly exposed to the hot venting fluid shows *Beggiatoa* mat overgrowth.

**FIGURE 11:**
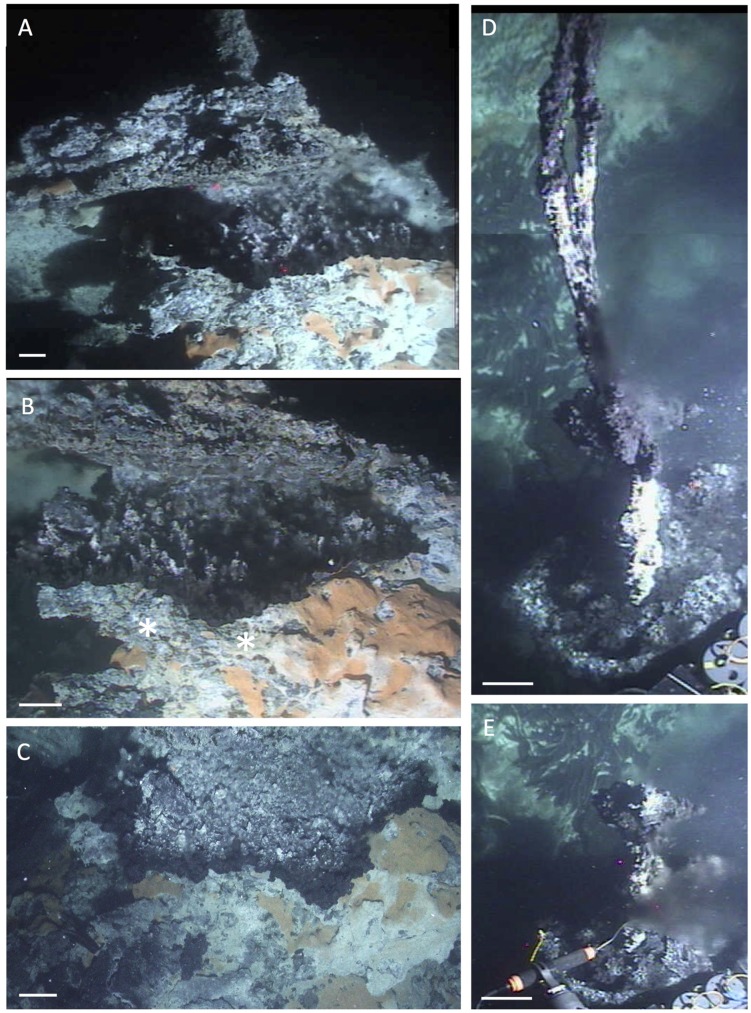
**Top of Rebecca’s Roost.** The large hydrothermal edifice “Rebecca’s Roost” was visited on *Alvin* Dive 4574, December 5, 2009. The scale bars correspond to 10 cm. **(A)** Composite framegrabber image of top venting orifice of Rebecca’s Roost as seen on *Alvin* Dive 4574, with shimmering fluid under flanges and mat-covered “lip” at the edge of the fluid source area. *Alvin* heading 163, depth 1989 m, GMT 20:23:11 and 20:22:41. **(B)** Framegrabber image of orange and white microbial mat right at the edge of the top venting orifice. *Alvin* heading 163, depth 1989 m, GMT 20:22:11. **(C)** *Alvin* external still camera photo showing “birds eye” view into the fluid source area, and mat-covered lip below. The mat-covered surface drops off near-vertically at the bottom of the photo. The photo was taken on the same *Alvin* dive shortly after photos **(A,B)**. Visual comparison of the orange *Beggiatoa* mats shows that two exposed rim pieces of the mat-covered lip (marked with asterisks in **B**) are missing here and were apparently broken off. **(D)** Composite framegrabber image of a peripheral thin chimney, viewed against the *Riftia*-covered walls of Rebecca’s Roost. Upper portion of the image: Alvin heading 214, depth 1989.8 m, GMT 20:27:11; lower portion: *Alvin* heading 213, depth 1990 m, GMT 20:26.41. **(E)** After the highly fragile chimney top was broken off, a vent fluid *in situ* temperature of 313.8°C was measured with *Alvin’s* high-temperature probe penetrating into the base of the friable chimney, marked by a jet of grayish, shimmering hydrothermal fluid. *Alvin* heading 214, depth 1989.8 m, GMT 20:31.42. Photographs courtesy of Woods Hole Oceanographic Institution, from RV *Atlantis* cruise AT 15-56.

Currently the largest hydrothermal edifice in the frequently visited hydrothermal sampling area of the southern Guaymas Basin, “Rebecca’s Roost” reaches a height of ca. 20 m from the seafloor; the large size of this structure precluded attempts to construct composite images. The images of the top (**Figures 11A–C**) show the colorless, shimmering venting fluid emerging from a diffusively venting zone marked by a gray mineral matrix, visible slightly below a fragile and broken outer crust covered with *Beggiatoa* mats that appears to encase the venting zone like a broken eggshell. Extremely thin and fragile chimneys on the flanks of the trunk provided a jet-like outflow for light-gray (not black) hydrothermal fluid into the surrounding seawater (**Figures 11D,E**). These friable structures break off easily and could be penetrated with *Alvin’s* high-temperature probe to measure the temperature of the hydrothermal outflow directly, here determined as 313.8°C (**Figure 11D**). These temperatures were close to those of hydrothermal fluids (315°C) originating from a shallow subsurface basaltic intrusion at the East Hill site, north of this sampling area; the East Hill fluids were among the hottest in Guaymas Basin, and the East Hill chimney sulfides showed an abiotic δ^34^S signature near zero, consistent with an abiotic, strictly hydrothermal origin (Peter and Shanks, 1992). By inference, the large edifice of Rebecca’s Roost may also function as the outlet for a hydrothermal flow path that does not allow for significant subsurface mixing and cooling.

**FIGURE 12:**
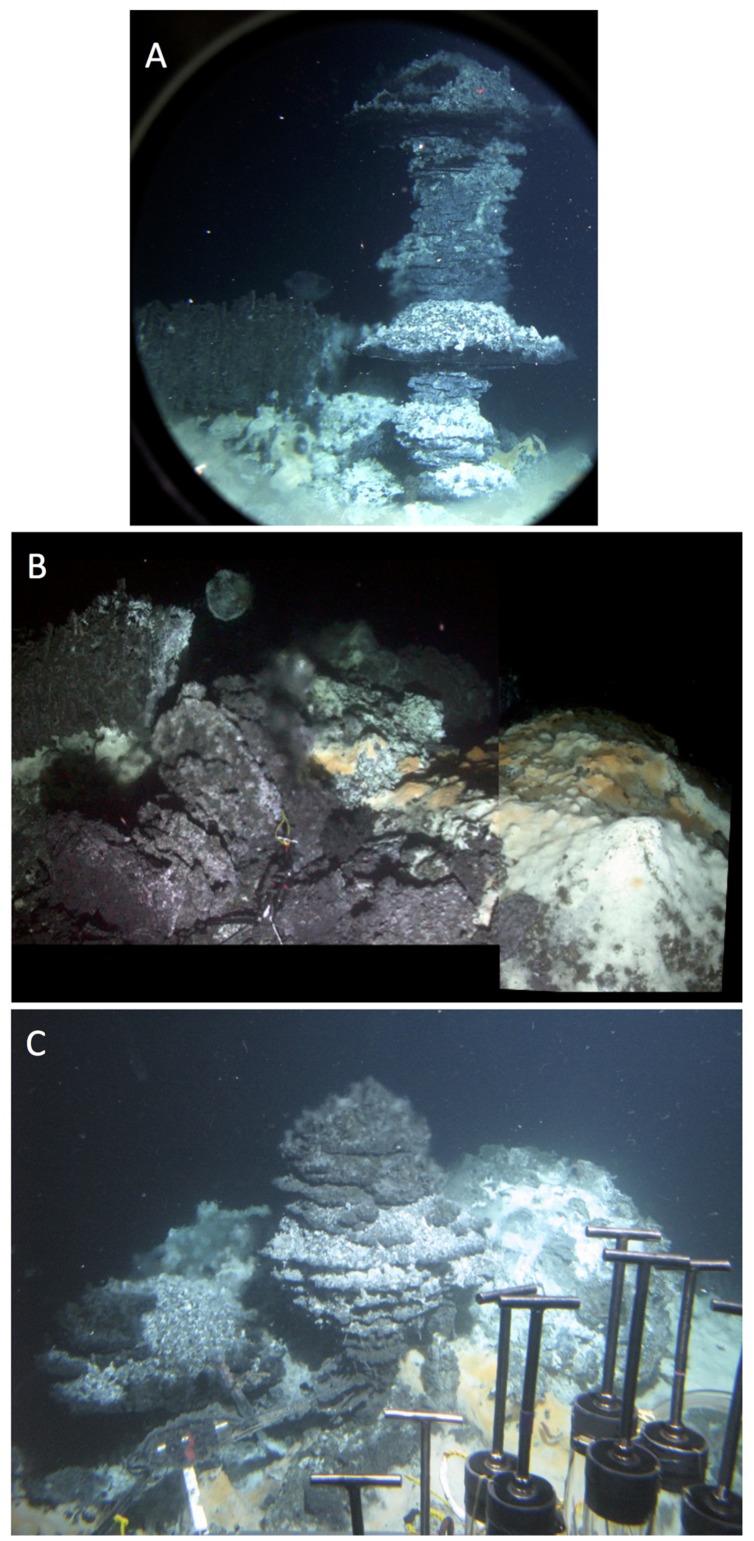
**Busted Mushroom.** Mushroom-like deposits on the top of the “Busted Mushroom” hydrothermal edifice. **(A)** Two “mushrooms” photographed on November 10, 2009 during cruise AT15-55 (*Alvin* dive 4555); the tree-trunk-like stem of a previously toppled mushroom can be seen to the left of the standing mushrooms. Comparable frame grabber images: *Alvin* heading 326, depth 1990 m, GMT 19:05. **(B)** A 2-day old fragile beehive grew within and around the cylindrical end of the array that was placed over the vent that fed the shorter mushroom in **(A)**. New material engulfing the eight thermocouples is visible at end of the titanium frame in the lower left of photograph, beneath schlieren caused by hot fluid flow; pieces of the fallen stem of the taller mushroom in **(A)** are located to the immediate left of the titanium frame, in front of the older fallen stem. Microbial mats in the foreground coat much of the top of the edifice, which was significantly larger than the portion photographed here on November 12, 2009 (*Alvin* Dive 4557, cruise AT15-55). Corresponding frame grabber image: *Alvin* heading 310, depth of 1989.5, GMT 20:42:12. **(C)** As observed on *Alvin* dive 4571, a 15-day-old mushroom and stem grew through an array deployed at a second vent opening, to the taller mushroom shown in **(A)**. Corresponding frame grabber image: *Alvin* heading 180; depth 1990 m, GMT 17:17.45. All photographs courtesy of the Woods Hole Oceanographic Institution, from RV *Atlantis* cruises AT15-55 and AT 15-56.

South of Rebecca’s Roost rises another tall but narrower hydrothermal edifice called “Busted Mushroom,” named after the mushroom-like edifices present on its top and material from toppled mushroom edifices (**Figure 12A**). Orange/white *Beggiatoa* mats coated the surfaces of the mound at the base of the mushroom stems (**Figure 12B**). A study of microbial colonization of active mushroom-like chimneys carried out on this mound revealed compositional differences in archaeal communities associated with very young (4-day) and older (72-day) chimney material that grew within and around arrays of eight thermocouples within a Titanium frame placed over the active vent (Pagé et al., 2008). These archaeal communities underwent a shift from autotrophic, CO_2_/H_2_-dependent hyperthermophilic methanogens colonizing the 4-day chimney material (predominantly *Methanocaldococcus* sp.) toward methylotrophic/acetoclastic methanogens (*Methanosarcinales*) and fermentative heterotrophic thermophiles (*Korarchaeota*, *Aciduliprofundales*) in the 72-day old chimney material. In 2009, additional arrays were deployed during cruise AT15-55 to further investigate the timelines of microbial colonization on new chimneys. Here, these experiments illustrate the extremely fast growth of hydrothermal chimneys within days. During Alvin dive 4555 on November 10, 2009, a small mushroom structure (foreground, **Figure 12A**) was razed and an array deployed to monitor time lines of mineral precipitation. Two days later, Alvin dive 4557 recovered this array and the fragile beehive chimney that had grown around it (**Figure 12B**). After several rounds of array deployments, the last array of cruise AT15-55 was deployed (November 17, 2009) over the orifice of the larger mushroom structure (which had fallen in the meantime), to be recovered on Alvin dive 4571 during the subsequent cruise (December 2, 2009). During this 15-day interval a mushroom edifice had grown through the array and the stem that developed had engulfed four of the eight thermocouples (**Figure 12C**). The chimney (mushroom stem) wall that developed within the array over the 15-day interval was similar in structure to the chimney walls recovered after 72 days in 2003; these were dominated by calcite and also contained variable amounts of barite, anhydrite, and metal sulfides (Pagé et al., 2008). Temperature and microbial data from the 2009 deployments were compared to data from 2003 to provide information about how the chimneys grow, what the temperatures were at the different places sampled, and to investigate compositional differences in archaeal and bacterial communities as functions of temperature and time (Reysenbach and Tivey, pers. comm.).

**FIGURE 13:**
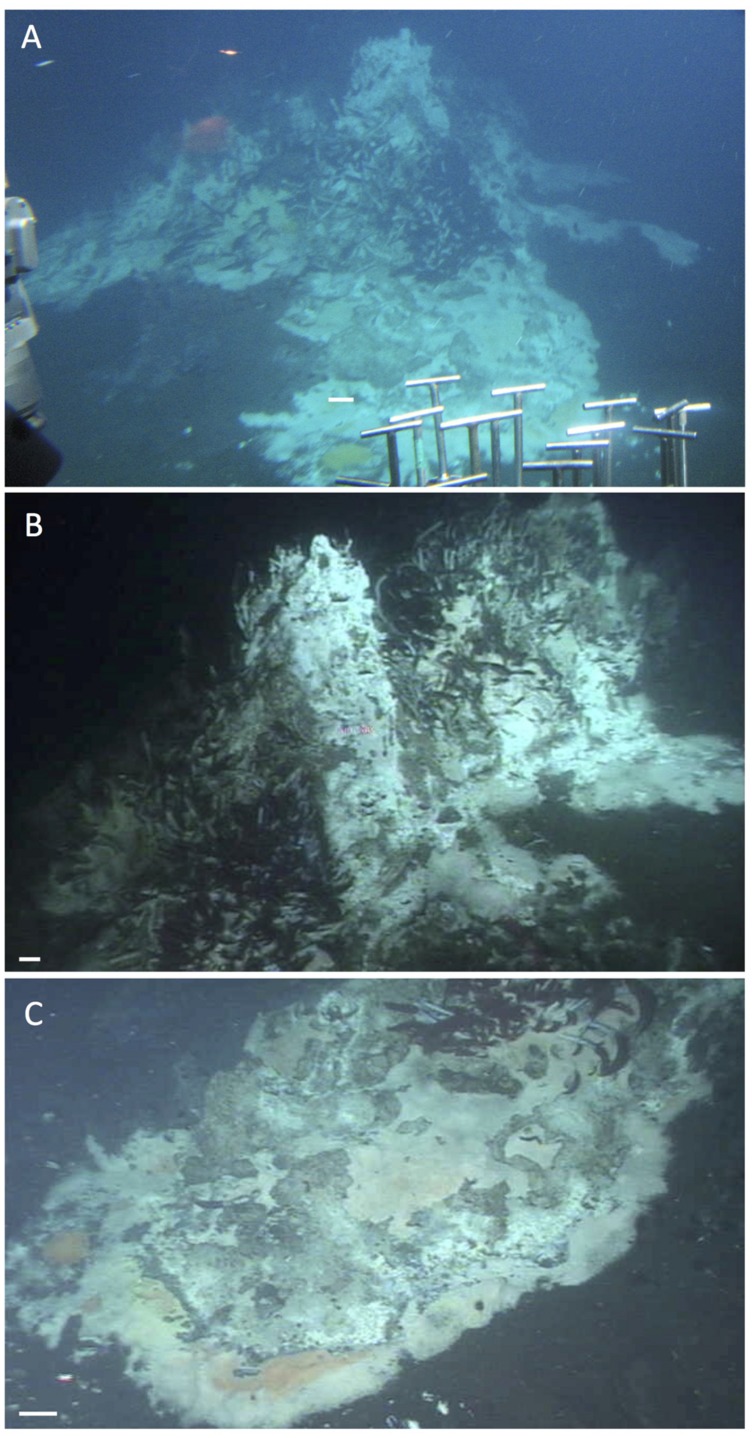
**Mat Mound, an extensive hydrothermal mound ca. 2.5 m high and several meters across.** The scale bar corresponds to 10 cm. **(A)** Still photo from inside *Alvin* approaching Mat Mound from the east during *Alvin* dive 4483 (December 6, 2008). The photo shows the multiple pinnacles, extensive *Riftia* overgrowth, and downward sloping microbial mats growing on the hydrothermal flanks and surrounding sediments of Mat Mound. **(B)** The central pinnacle of Mat Mound in a framegrabber image from the subsequent dive. *Alvin* heading 285, depth 2002 m, GMT 21:45:18, dive 4484, December 7, 2008. **(C)** Extended slope of Mat Mound with *Riftia* colony higher up, and patchy white and orange *Beggiatoa* mats covering the lower slope and the sediment contact zone. *Alvin* heading 238, depth 2003 m, GMT 18:40:10, dive 4484, December 7, 2008. All photographs courtesy of the Woods Hole Oceanographic Institution, from RV *Atlantis* cruise AT 15-40.

A smaller hydrothermal structure, “Mat Mound” (**Figure 13A**), was investigated in detail with *Alvin* dives in December 2008. “Mat Mound” combined features of hydrothermal edifices such as “Big Pagoda” and “Rebecca’s Roost,” for example the steep, diffusively venting walls that were overgrown by *Beggiatoa* mats and young *Riftia* colonies (**Figure 13B**), with the characteristics of hydrothermal mounds, such as the extensive basal slopes that are surrounding the structure like a ring of talus debris originating from the steep walls. Some of these basal slopes are in themselves hydrothermally active (**Figure 13C**). Temperature point measurements showed a moderate thermal regime of cool temperatures on the mound walls (6 to 15°C), hot temperatures near the base of the mound (50–100°C), and cooler temperatures at the surface of the surrounding sediments nearby (10°C; Dowell et al., 2016). No evidence for channelized hydrothermal flow or a chimney-like orifice was found, and diffusive venting through the mound mineral matrix appeared to predominate. The contact zone between the mound and the surrounding sediment harbored thick microbial mats and the steepest temperature gradients, at and above 100°C at 40 cm sediment depth (Dowell et al., 2016). Mat-covered sediments surrounding “Mat Mound” were characterized geochemically and microbiologically; they turned out to be strongly sulfidic and reducing, and contained seawater sulfate coexisting with high methane concentrations, thus providing a suitable habitat for heat-tolerant, sulfate-dependent, methane-oxidizing microbial communities (Dowell et al., 2016). The bare sediments at a short distance (∼1 m) from the mound contained only minimal concentrations of porewater sulfide and methane, and the *in situ* temperatures down to 40 cm sediment depth were reduced to a range between 3 and 5°C; obviously, the hydrothermal gradients had dissipated a short distance from the mound.

**FIGURE 14:**
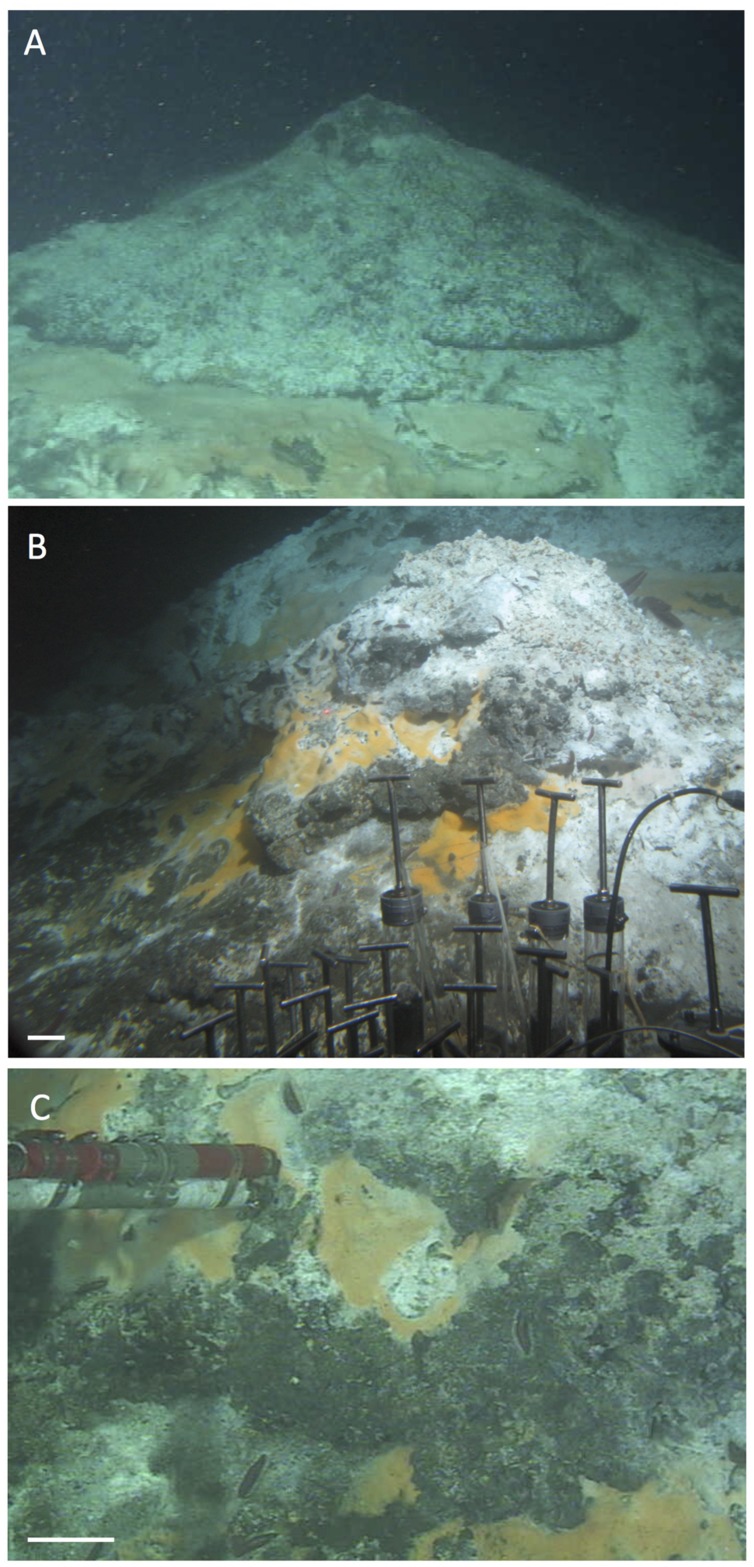
**Wonder Mound.** This gently sloping hydrothermal mound with a single peak was overgrown with microbial mats but only few clusters of *Riftia*. Images are from *Alvin* dive 4562, November 23, 2009. The scale bars correspond to 10 cm. **(A)** Frame grabber image of the top of Wonder Mound; the peak is surrounded with lobed structures that could be incipient flanges, hugging the contours of the mound instead of protruding into the water. *Alvin* heading 108, depth 1997 m, GMT 22:09:31. **(B)** External still image of mound flanks with orange *Beggiatoa* mats. *Alvin* heading 108, depth 1997 m, GMT 22:06:14. *In situ* temperatures of 24°C, 8°C and 10°C, respectively, were measured with the high-temperature probe in surficial orange mats in the center of this image (GMT 22:06:30 to 22:09:01). **(C)** Frame grabber image of slurp gun sampling of orange *Beggiatoa* mats from the base of Wonder Mound. *Alvin* heading 108, depth 1997 m, GMT 22:13:01. All photographs courtesy of the Woods Hole Oceanographic Institution, from RV *Atlantis* cruise AT 15-56.

Nearby, an even larger mound was visited on Alvin dive 4562 in November 2009, and termed “Wonder Mound” for its imposing and massive appearance, with an estimated height of ca. 4–5 m and a diameter of more than 10 m based on *in situ* observation (**Figure 14**). In contrast to “Mat Mound,” *Riftia* colonies were either absent or reduced to small clumps (visible in the bottom left corner of **Figure 14A**). Orange and white *Beggiatoa* mats were well developed (**Figure 14B**) and could be harvested from the surface of the mound with *Alvin’s* suction device, called the “slurp gun” (**Figure 14C**). The moderate *in situ* temperature regime on the mat-covered surface (8–24°C at three different spots in the orange *Beggiatoa* mat in **Figure 14B**) is compatible with diffuse venting of mixed fluids that permeate the outer walls of this mound. The pointed top of “Wonder Mound” and the thick flange-like lobes that followed contour lines around the peak and emitted shimmering water and/or rising particles, suggested a hydrothermal hot spot characterized by strong diffusive venting (**Figure 14A**).

**FIGURE 15:**
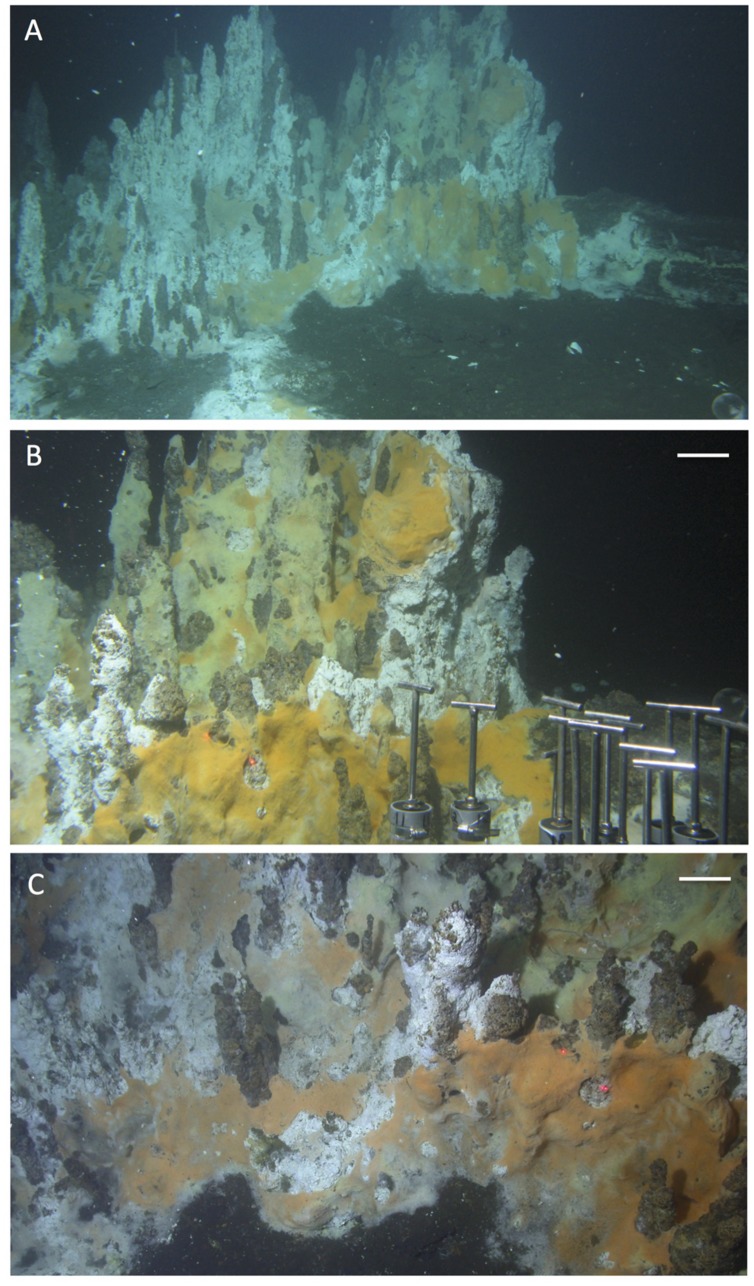
**Notre Dame, a multi-chimney hydrothermal edifice, ∼3 m high.** The site was named in reference to the soaring vertical lines and visual splendor of gothic cathedrals. All pictures were taken with the external still camera during *Alvin* dive 4573, December 4, 2009, depth 2012 m. Based on the framegrabber records, the corresponding *Alvin* headings are in the range of 116 to 133. **(A)** Overall view of the hydrothermal edifice, heading approximately Southeast, GMT 19:36:53. **(B)** Close up of microbial mat network on the tall chimneys; GMT 19:38:35. **(C)** Base of mound and bare sediment, GMT 19:37:26. The orange and yellow mats appear to overgrow the bare surfaces of the chimney-like edifices and their patchy white coating, possibly sulfur precipitates. All photographs courtesy of the Woods Hole Oceanographic Institution, from RV *Atlantis* cruise AT 15-56.

The most visually dramatic hydrothermal edifice found during RV *Atlantis* cruises AT15-40 and AT15-56 was a wall of vertical hydrothermal chimneys, first observed during *Alvin* dive 4573 on December 4, 2009 (**Figure 15A**). This ∼2 m high structure, termed “Notre Dame” by the observers to prevent confusion with the “Cathedral Hill” location sampled previously on dive 4565, was almost entirely covered with white, yellow, and orange *Beggiatoa* mats (**Figure 15B**). The mats colonized exclusively the chimneys but ended exactly at their base, and did not extend into the surrounding sediments (**Figure 15C**). Due to time limitations, no *in situ* temperature measurements were made.

## Discussion

### Subsurface Context of Hydrothermal Features

The diverse hydrothermal sediments, mats, mounds, and chimneys documented here show distinct distribution patterns across the Guaymas Basin seafloor that are ultimately linked to subsurface hydrothermal circulation and heat sources. Early Deep-tow sonar surveys of the central southern Guaymas trough indicated several sills buried at shallow depths (Lonsdale and Becker, 1985). Close to the hydrothermal area surveyed here, a shallow sub bottom intrusion (<100 mbsf) was interpreted as a thin sill; the approximate positions of this and a similar sill nearby (based on Figure 2 in Lonsdale and Becker, 1985) were revised, extrapolated and combined into a continuous boomerang-shaped sill, the southernmost of three sills that are lined up in the center of southern Guaymas Trough (mapped in Peter et al., 1990 and Peter and Shanks, 1992). If these inferred sill positions are correct, the cluster of hydrothermal mounds and microbial mats sampled during *Atlantis* and *Alvin* cruises AT15-40 and AT15-56 would trace the southeastern arm of the boomerang-shaped, southernmost sill (**Figures 1A,B**), and confirm previous conclusions on the relationship between hydrothermal features and sill boundaries (Lonsdale and Becker, 1985). As a corollary, the hydrothermal fluids that migrated to the sediment surface within this sampling region would most likely originated from the same local hydrothermal circulation system linked to this sill. Some indirect geochemical evidence supports this scenario. The δ^13^C baseline values of hydrothermal methane from hot sediments in this region (>150°C, to exclude biological imprint) form a tight cluster from –39.09 to –43.18aaaa; (McKay et al., 2015) that contrasts with the more variable δ^13^C values between –51 and –43aaaa; for hydrothermal methane reported previously from different sites in Guaymas Basin (Welhan, 1988). Interestingly, lighter δ^13^C values of –50.8 and –45.1aaaa; were obtained from hydrothermal fluid samples collected during *Alvin* dives 1169 and 1175 in the “North Hill” region of the southern Guaymas trough, several miles to the north near the northernmost of the three mapped sills; whereas a matching δ^13^C value of –43.2aaaa; was obtained for a hydrothermal fluid sample collected on dive 1173 within the same sampling area as surveyed here (Welhan and Lupton, 1987). A wider-ranging chemical and isotopic survey of hydrothermal fluids in Guaymas Basin could obviously extend these initial data and allow the development of a spatially resolved regional database that could be useful in identifying and mapping subsurface hydrothermal circulation patterns. The hypothesis of a shared subsurface methane source for the commonly visited sampling area on the southern sill would be consistent with a localized, sill-to-surface hydrothermal circulation pattern, as proposed after Deep-Sea Drilling Program Leg 64 provided the first view of the Guaymas Basin subsurface (Kastner, 1982). The approximate depth range of this circulation pattern could be inferred from the depth of the middle sill mapped in **Figure 1** that was drilled twice during DSDP Leg 64. At these two adjacent locations (DSDP holes 477 and 477A, marked by red dots), the underlying sill extended from depths of 58 to 105.5 mbsf and 32.5 to 62.5 mbsf, respectively (Shipboard Scientific Party, 1982).

While the mat-rich area also includes diffusively venting hydrothermal mounts, the largest hydrothermal edifices characterized by channelized fluid flow (Big Pagoda, Rebecca’s Roost) were located farther north (**Figure 1B**). This transition from mat-dominated hydrothermal sediments and hydrothermal mounds without channelized flow, toward actively venting chimneys and flange-lined chimney tops coincided with a north-trending increase in heat flux (Fisher and Becker, 1991) that ranged from 100 to 300 mW per m^2^ southwest of the mat-dominated hydrothermal field, toward 300–600 mW per m^2^ in the area with large hydrothermal chimneys to the North (**Figure 1B**). A comparison of the heat flow map of the southern Guaymas trough (Figure 2 in Fisher and Becker, 1991) and of the hydrothermal features and subseafloor sills compiled in **Figure 1A** shows that the areas of highest heat flux coincide broadly with the two mapped sill areas positioned further northeast along the trough axis. We also call attention to the observation that the largest hydrothermal features with channelized fluid flow (Big Pagoda, Rebecca’s Roost) appear to be located over the central portion of the underlying sill, not on its periphery, suggesting localized hydrothermal flow through a fault line within the sill; this possibility was noted previously (Lonsdale and Becker, 1985).

To the best of our knowledge, the hydrothermal areas to the north of the commonly visited sampling area are currently neglected by *Alvin* dives, at least since early surveys of Guaymas Basin in the 1980s (Lonsdale and Becker, 1985); published sampling records in microbiological studies of Guaymas Basin indicate that the same well-known dive targets in the region described here are visited repeatedly by our own and by other microbiologically oriented cruises (Campbell et al., 2001, 2013). To obtain a greater diversity of hydrothermal samples from both the northern and southern Guaymas Basin troughs and their ridge flanks (Lizarralde et al., 2011), and to document a fuller range of hydrothermal habitats, new survey tools, such as the autonomous ROV *Sentry* appear highly promising.

An improved survey of Guaymas Basin would also provide a greater range of sampling sites to address open questions, such as fluid and gas transport through different types of hydrothermal sediments, and the overall budget of carbon, sulfur, nitrogen and other elemental fluxes across the sediment/water interface. Previous work appears to be limited to heat flow surveys (Williams et al., 1979; Lonsdale and Becker, 1985; Fisher and Becker, 1991), basin-wide studies of Helium-3 and manganese accumulation (Lupton, 1979; Campbell et al., 1988), and initial estimates of hydrothermal carbon mobilization and loss from the overall sediment volume affected by sill intrusion (Lizarralde et al., 2011); therefore, quantitative investigations of hydrothermal transport in Guaymas Basin represent a wide-open research field. Quantifications of microbially catalyzed biogeochemical processes in hydrothermal sediments of Guaymas Basin have so far focused on sulfate reduction (Elsgaard et al., 1994; Weber and Jørgensen, 2002; Meyer et al., 2013) and to a lesser extent on nitrate reduction (Bowles et al., 2012). These and other microbial activities could be linked to microbial gene expression studies on the level of specific functional genes, as demonstrated for archaeal methane oxidation in Guaymas Basin sediments (Biddle et al., 2012), or on the level of metatranscriptomes as shown for archaeal nitrification in hydrothermal plumes (Baker et al., 2012). Temperature regimes permitting, microbial processing of hydrothermal carbon and energy sources is not limited to the surface. Ultimately, recovering subsurface sediments and sill sections by deep coring and drilling would yield significant scientific returns about biological, chemical and hydrothermal gradients beyond the surficial sediment layers in Guaymas Basin (Teske et al., 2014).

## Author Contributions

AT compiled the figures, did the data archaeology, and wrote the paper; DD performed and plotted the *in situ* microprofiler measurements; LM compiled and plotted the heatflow probe temperature profiles; JB performed the 2008 and 2009 observations at Marker 6; MT performed the mineralogical analyses at Busted Mushroom and contributed the photos of this site; DH, KL, ML, HR, DA, and HM contributed *in situ* observations and geochemical and thermal measurements of different hydrothermal sediments and mats, BM mapped the hydrothermal seafloor locations in the context of subsurface sill and heatflow locations.

## Conflict of Interest Statement

The authors declare that the research was conducted in the absence of any commercial or financial relationships that could be construed as a potential conflict of interest.

## Footnotes

1 www.whoi.edu/page.do?pid=11042

2 http://www.marine-geo.org/references/descriptions/LocalXY.php

3 http://www.whoi.edu/marine/ndsf/utility/NDSFutility.html
