# Supplementary material for: The Guaymas Basin Hiking Guide to Hydrothermal Mounds, Chimneys, and Microbial Mats: Complex Seafloor Expressions of Subsurface Hydrothermal Circulation
Source: Front Microbiol. 2016 Feb 18;7:75. doi: 10.3389/fmicb.2016.00075 (PMC4757712; doi:10.3389/fmicb.2016.00075)
Supplement: Supplementary file 1 [file Presentation_1.PDF]

Supplementary Online Figures

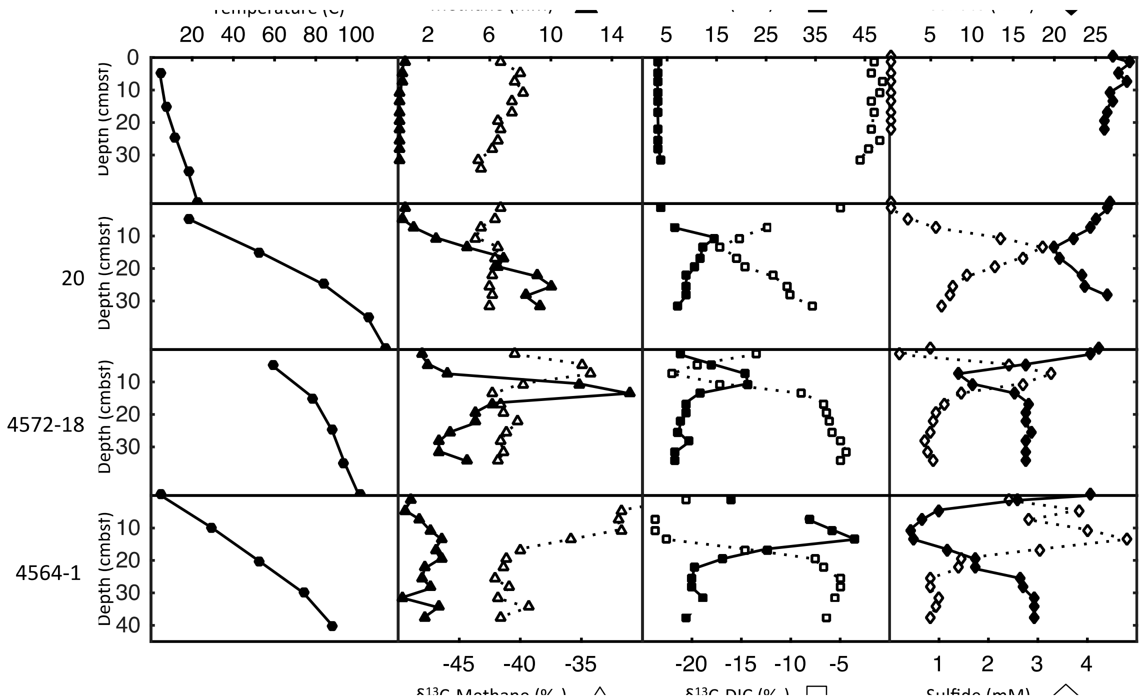

Figure S1. Porewater geochemical and thermal profiles for four coring positions from the mat shown in Figure 2C. Core 4572-14 was collected from the bare sediment outside of the mat near the cool heat flow profile marked in blue; core 4572-20 was collected in the white mat area, near the red heat flow profile to the right of the orange center; core 4572-18 was collected near the pale green heat flow profile in the center of the orange mat; and core 4564-1 was collected on the border of the orange and white mat area, approx. halfway between the yellow and green heat flow profiles. Data for cores 4572-14, 20 and 18 were replotted from McKay et al., 2012; core 4564-1 was newly included.

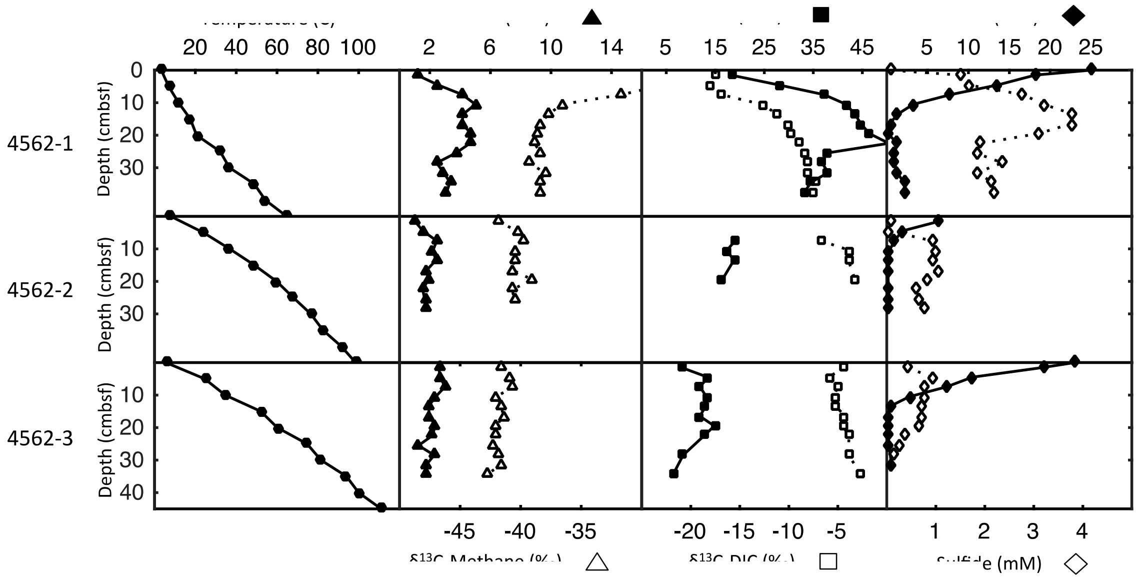

Figure S2. Porewater geochemical profiles for three Megamat cores, representing the gradient from cooler sediment without mat cover towards hotter mat-covered sediments shown in Figure 5A. Core 4562-1 was taken near the cooler dark-blue heatflow profile; core 4562-2 was taken near the warmer heatflow profile marked in dark green, and core 4562- 3 was taken near the hottest heatflow profile in orange.

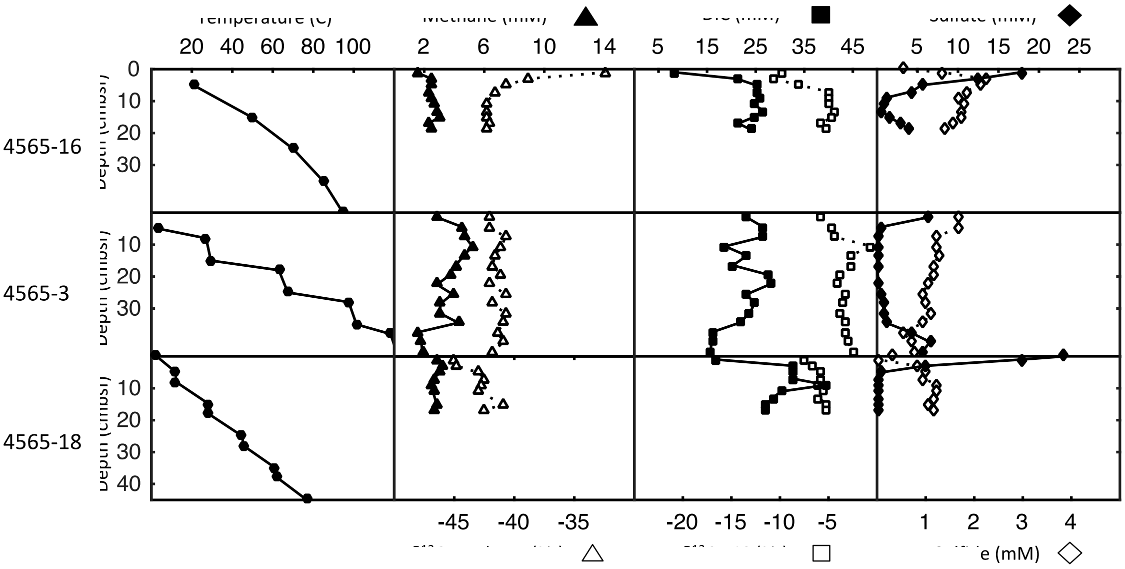

Figure S3. Porewater geochemical profiles for Cathedral Hill area, as shown in Figure 5B. Cores 4565-16 and 4565-3 were taken from hot sediments between the maroon- and orange-colored heatflow profiles; core 4565-18 was taken near the more temperate heatflow profile marked in yellow.
